# Illumina reads correction: evaluation and improvements

**DOI:** 10.1038/s41598-024-52386-9

**Published:** 2024-01-26

**Authors:** Maciej Długosz, Sebastian Deorowicz

**Affiliations:** Faculty of Automatic Control, Electronics and Computer Science, Silesian University of Technology, 44-100 Gliwice, Poland

**Keywords:** Genome assembly algorithms, DNA sequencing, Next-generation sequencing, Data processing, Software

## Abstract

The paper focuses on the correction of Illumina WGS sequencing reads. We provide an extensive evaluation of the existing correctors. To this end, we measure an impact of the correction on variant calling (VC) as well as de novo assembly. It shows, that in selected cases read correction improves the VC results quality. We also examine the algorithms behaviour in a processing of Illumina NovaSeq reads, with different reads quality characteristics than in older sequencers. We show that most of the algorithms are ready to cope with such reads. Finally, we introduce a new version of RECKONER, our read corrector, by optimizing it and equipping with a new correction strategy. Currently, RECKONER allows to correct high-coverage human reads in less than 2.5 h, is able to cope with two types of reads errors: indels and substitutions, and utilizes a new, based on a two lengths of oligomers, correction verification technique.

## Introduction

Despite the growing popularity of newer technologies, Illumina sequencers are still broadly used, producing huge amounts of data. In general, Illumina sequencing reads are of good quality but still some fraction of bases are recognized wrongly, which complicates downstream analyzes. Therefore, de novo assemblers, read mappers, variant callers, etc., are designed having in mind that sequencing errors are possible. Nevertheless, handling of errors in them is usually simple, as this is not the main task of such tools.

Over the years, we see a constant interests in designing specialized sequencing error correctors^1–10^. Some attempts to evaluate their performance can be found in Refs.^11–14^. Typically, but not completely, efficacy of the read error correction algorithms (and in effect—meaningfulness of they utilization) is analyzed with a group of methods:

- comparing simulated (i.e., generated with a computer) erroneous reads processed by a tested algorithm with their original (error-free) versions,
- measuring an impact of the real (non-simulated) reads correction on the quality indicators (like contig length) in de novo assembly,
- examining whether after the correction the real reads map better (i.e., with lower number of mismatches, or even exactly) to a reference genome.

The aforementioned methods have pros and cons, however, they do not tell us what is an impact of correction on any of high-scale processes, like variant calling (VC). Although VC is based on reads mapping, subtle differences in mapping results not necessarily lead to calling wrong variants. Thus, there is no direct translation of the fraction of corrected simulated reads (or even real reads) to how many variants will be called wrongly. One of our goals is to fill this gap. In the paper, we focus on detection of short variants, i.e., single nucleotide polymorphisms (SNPs) or short insertions and deletions (indels).

To the best of our knowledge, that problem was raised in the literature only three times. In Ref.^9^ the authors analyzed impact of correction on two human chromosomes in terms of sensitivity, precision, and F1-score. In Ref.^15^ the values of sensitivity and precision of number of detected variants were measured for very short *E. coli* reads, but only for the Quake algorithm, which is currently rather outdated. The reads of *H. sapiens* were corrected with Quake and VC was performed, but only to illustrate an increase of number of called variants. In Ref.^7^ *C. elegans* dataset was used to estimate the number of false positive SNPs. This was made by comparing number of variants called for contigs generated for corrected reads and a set (treated somewhat like a ground truth) of variants called for uncorrected reads. None of the above assays concerned indels.

Some preliminary attempts to the VC-based evaluation of sequencing data correctors were also performed in our previous work^16^. In this paper, we extend that assay to show an impact of various correction algorithms to VC. We use two differently-sized and differently-characterised organisms, i.e., *A. thaliana* and *H. sapiens*, for two complementary criteria: SNP and indel calling quality. The experiments were done on a wide range of sequencing depths to illustrate its impact.

Moreover, we also test the influence of correction on de novo assembly. This is made on Illumina NovaSeq sequencing reads. To the best of our knowledge, such reads were not used in another papers, albeit they have different characteristics than often utilized ones, like from HiSeq and MiSeq machines. In NovaSeq sequencers, the number of possible values of symbols quality scores is reduced to 4 (in contrast to 8 or about 40 in older machines) and reads quality is affected by another defects, listed in Table 1 in Supplementary material. Many correction algorithms use that values as a suggestion which regions of the read could be impacted by errors, hence decrease of its scope may cause algorithms to fail. In Ref. ^9^ an impact of lossy scores compression on SAMDUDE corrector was examined, but in a context of VC only.

In the previous works, we proposed our own Illumina data correction algorithm —RECKONER^17,18^. In this paper, we present its new version, introducing a number of enhancements. We added an option to explicitly correct indel errors (in contrast to the most common in Illumina reads, substitution errors), which are not handled by a majority of algorithms. We also designed a method of guiding correction process, based on an idea of long subwords of reads of length $k'$ ($k'$-mers), performed along with correction based on typical, short *k*-mers, i.e., $k' > k$. It detects, if the corrected reads consist of $k'$-mers present at least one time in a whole set of reads. The idea turned out to be especially beneficial for preparing reads to be de novo assembled. The aforementioned changes enforced adapting the other concepts of the algorithm, including representing the current state of the read correction process in a memory, and the method of rating different ways of the specified read correction (correction paths).

## Results

Table 1 contains accession numbers of reads, reference genome identifiers, and versions of ground truth VC sets used in the experiments.

**Table 1:** Read datasets and genomes used in the experiments.

Organism	Read accession numbers	Reference genome	VC ground truth
*Homo sapiens*	ERR174324 ERR174325 ERR174326 ERR174327	GRCh38	NA12878 (HG001), v. 3.3.2
*Arabidopsis thaliana*	SRR1945754	TAIR10.1	Intersection 6904, v. 3.1
*Chlorella vulgaris*	SRR9478717	GCA_008119945.1	–

**Figure 1:**
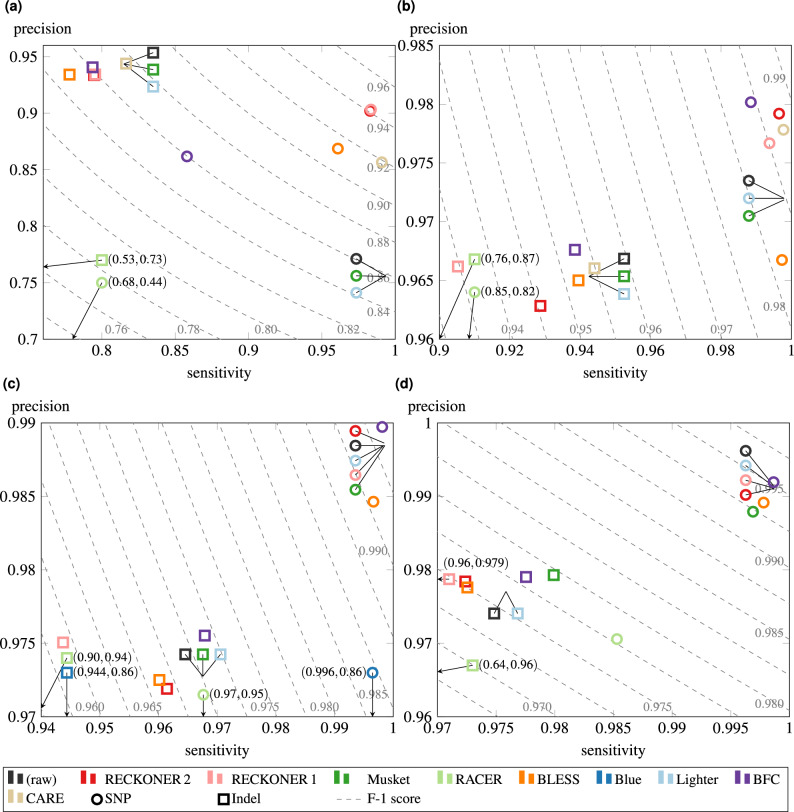
F1-score for *H. sapiens* VC—Strelka and hap.py. Plot (**a**) shows results for $15\times$ sequencing depth read sets, corrected with different algorithms, (**b**) for $30\times$, (**c**) for $45\times$, (**d**) for $60\times$. The plot idea is based on Ref.^19^.

Verifying VC for human reads was performed in terms of ability to call both SNP and short indel variants, measured with their F1-score, for HG001 dataset from GIAB collection^20^. The VC results for various sequencing depths are shown in Fig. 1. The protocol of evaluation was: correct reads with an examined algorithm, map them with BWA-MEM^21^, call the variants with Strelka^22^, and evaluate them with Haplotype VCF comparison tools (hap.py)^23^. The separated values of all the measures for hap.py, the sensitivity and precision results for variants obtained with a caller DeepVariant^24^, all the results for another evaluating tool, Syndip^25^, genotype concordance measures computed with GATK ^26^, and the results obtained separately for homo- and heterozygous variants, were included in the Supplementary material. We performed the experiments on a group of correctors described in Refs.^1–10^, including the new version of RECKONER (RECKONER 2). For a reference we included also uncorrected (raw) reads and the previous version of RECKONER^18^ (RECKONER 1).

In all the VC experiments, we excluded Fiona^4^, as it changed corrected read quality scores by replacing them with the smallest possible values. It caused Strelka to generate no variants. Karect^8^ and SAMDUDE were not able to perform any correction (respectively, due to exceeding 72 hours time limit, and a runtime crash). Blue^5^ and CARE^10^ have crashed for some depths due to a lack of memory.

Impact of the sequencing depth is as expected. For the small ones, the results were worse than for the bigger ones. For SNPs detection results obtained for different algorithms are similar. The outstanding results are for RACER^2^, which loses in all the cases, and BLESS, CARE and, especially, RECKONER 2, which outstrip the competitors for the depth of $15\times$. It shows, that even for such a low amount of data, correction could cause the quality boost vs. the uncorrected reads. For higher depths, the differences are tiny. In the case of indels detection, the results more vary. Especially, RACER and Blue (for single successful outcome) negatively outstand, but for the smallest depth also RECKONER 2, BLESS^3^ and BFC^7^ worse the results. However, it should be mentioned, that calling the indels is eligible for higher sequencing depths, and for depths of $45\times$ and $60\times$ all the algorithms offer similar results, with a slight Musket^1^ advantage. We can also notice that the new, indel-aware mode of the current version of RECKONER 2 improves the F1-score by about 0.011 over RECKONER 1.

It has to be mentioned, that results for Lighter^6^ and Musket were obtained for extremely low values of *k*-mer length, c.a. 11–13. Such parameters allowed to obtain the best results for them, but at the expense of introducing almost no corrections. Results for other values are shown in Supplementary material.

**Figure 2:**
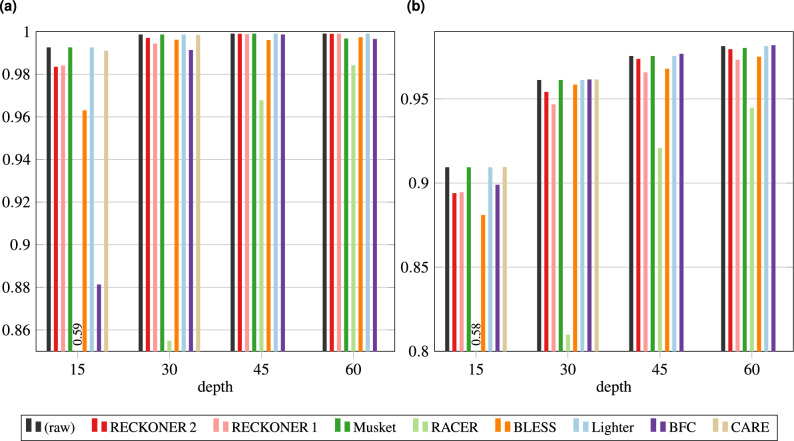
F1-score for *H. sapiens* VC—DeepVariant and hap.py. Plot (**a**) shows VC sensitivity for SNPs, performed with reads corrected with different algorithms, (**b**) for indels.

Selected results of experiments with DeepVariant variant caller are presented in Fig. 2. They are a bit surprising and disappointing as it looks that DeepVariant works best for uncorrected (raw) data. The best results among correctors are for Musket and Lighter that make no corrections and effectively the caller works on raw data. From the opposite side, BFC (one of the best algorithms) significantly hampers DeepVariant work, especially for low coverages. The behaviour of RECKONER 2 is in the middle, for coverages $45\times$ and $60\times$ it deteriorates the results a little.

Unfortunately, it is unknown how the caller makes the decisions, so we can only speculate about the reason of this phenomenon. We suspect that this is a result of selection of training data, which were raw (uncorrected) reads. Every corrector introduces some changes that could form some hidden patterns. This means that the characteristics of corrected data are different than of raw reads. In the learning stage, DeepVariant probably discovered some error patterns in raw data, but after the correction they could be destroyed and new patterns could be introduced by the correctors. These are, however, only speculations. To verify them, one should design an experiment in which a lot of raw reads were corrected by various tools. Then, such corrected reads should be used by DeepVariant in the learning stage. After that, the modified DeepVariant should be used to call variants to see whether this way we could improve the variant calling quality. Such an experiment would be large and we were not able to perform it in the paper.

**Figure 3:**
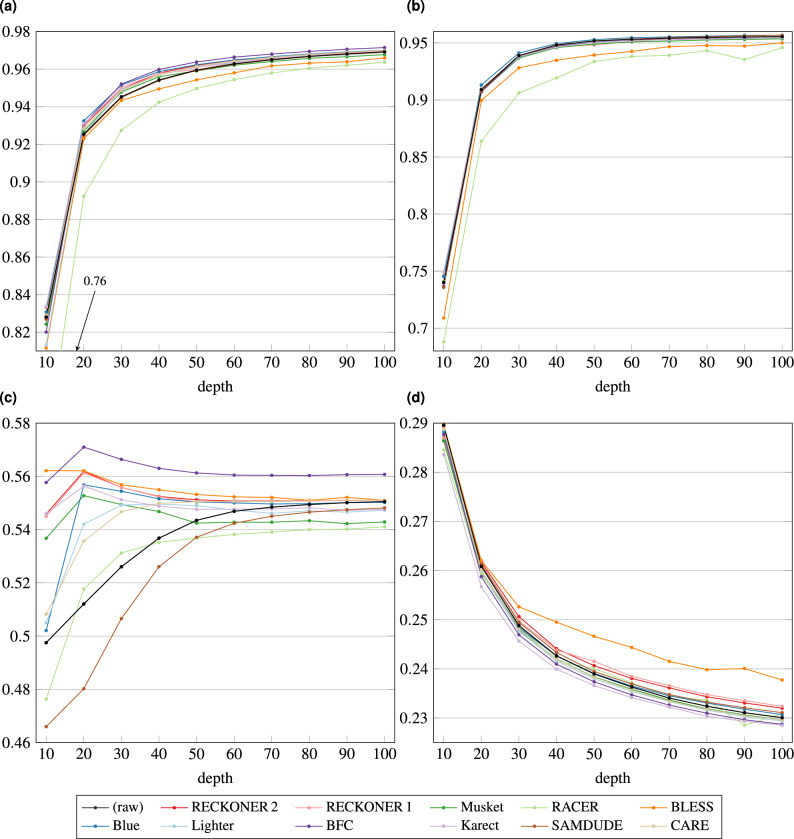
Results for *A. thaliana* VC—Strelka and hap.py. Plot (**a**) shows VC sensitivity for SNPs, performed with reads corrected with different algorithms, (**b**) for indels, (**c**) precision for SNPs, (**d**) for indels.

To observe how the correctors perform for other species, especially with shorter genomes, but with higher density of variants we used *A. thaliana* datasets from the 1001 Genomes Project (1001GP)^27^. This project contains huge number of read datasets with associated variants files, which can act as a ground truth. Experiments for *A. thaliana* were performed in the same way as for *H. sapiens*, excluding usage of confident call regions (unavailable for *A. thaliana*) and evaluating variants just with hap.py.

The VC sensitivity and precision for *A. thaliana* reads, for different sequencing depths are shown in Fig. 3. All the correctors succeeded, however the impact of sequencing depth is quite surprising here. In the case of sensitivity, as expected, a quality of variant calling increased along with the growing depth. However, precision for SNPs in many cases increases for moderate depth, then gradually decreases. For indels the decrease occurs in the whole range of the depth. Moreover, the values of both (SNP and indel) F1-scores are clearly lower than in the case of *H. sapiens*. The huge difference between sensitivity and precision values caused, that we presented them separately (F1-score results are shown in Fig. 7 in Supplementary material).

There are several possible explanations of that phenomena. First, the confident call regions are no available for this species. Second, the variant density is higher in *A. thaliana* than in *H. sapiens* which makes the correction problem much harder. The correctors are based on *k*-mer analysis and a single *k*-mer usually covers 0 or 1 variants for human data and more than 1 for *A. thaliana*, which makes the distinguishing between error and variant much harder. Third, the direct reason of the precision degradation (i.e., number of false positive variants increase along with the sequencing depth) is a used method of VC evaluation. As a reference we used a “model” variant set from the 1001GP, which is a result of a VC performed with two pipelines and a careful filtering, but this is not a typical, high-quality ground truth. Increasing of the depth causes Strelka to generate more variants, which is an appropriate behaviour, as it causes an amendment of sensitivity. Many of the detected variants are not present in the model set, hence the precision decreases. Filtering of the Strelka results, similar as 1001G authors did, is not a straightforward solution, because Strelka generates variants with non-standard quality scores, different from the standard Phred range of ca. 0–40. However, there is no warranty, that those additional variants are incorrect. To solve the matter we tried many ways of variant filtering and redeveloping the model strategies (results not included), however, none of them succeeded, i.a., due to not typical variants quality scores generated by Strelka.

Nevertheless, the results provide some useful information. The reads correction in most cases allows to increase the number of proper variants called, especially for SNPs. It is proven by an increase of sensitivity, which is actually visible just for SNPs (except for BLESS and RACER, which cause decrease of sensitivity, both for SNPs and indels). In most cases, the indel calling sensitivity is very similar for different correctors and the raw reads. The obvious thing is also a significant reduction of false positive SNPs for small values of the sequencing depth, except for RACER and SAMDUDE, manifesting itself by increase of precision. For indels the only corrector resulting in a clear precision difference from the raw reads is BLESS. Its output reads caused the lowest number of false positive variants. In summary, most of correctors may allow to generate more proper variants, however the method of the evaluation does not allow to draw the precise conclusions, hence it needs to be further explored.

**Figure 4:**
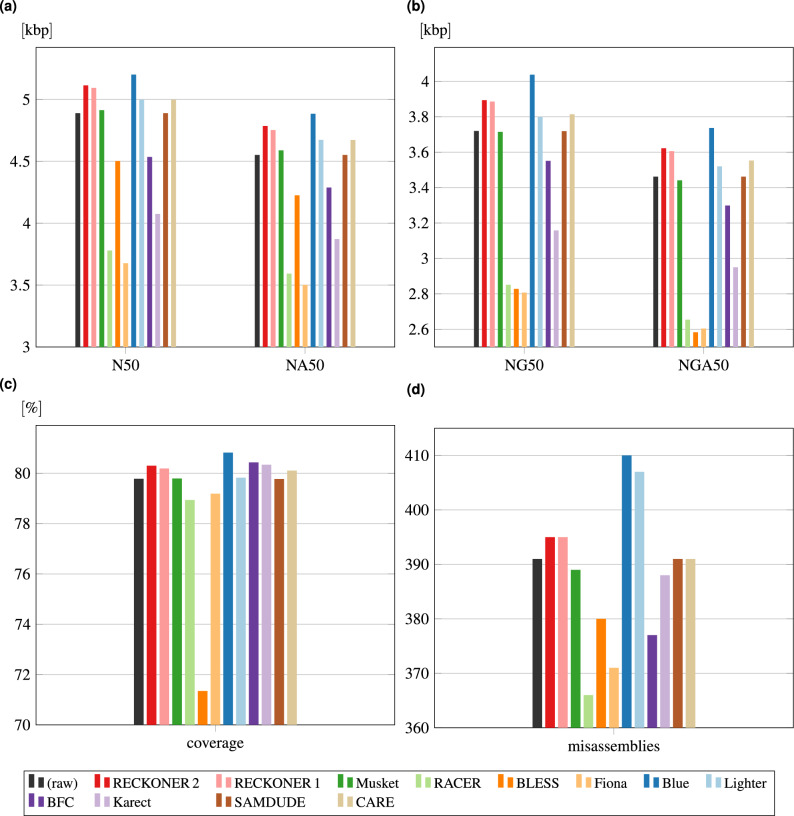
Assembly quality of NovaSeq *C. vulgaris* reads—Minia. Plot (**a**) shows N50 and NA50 measures for de novo assembly of the reads corrected with different algorithms, (**b**) NG50 and NGA50, (**c**) genome coverage, (**d**) number of missassemblies.

To verify a correction efficiency of NovaSeq data, we performed de novo assembly with Minia^28^ for *C. vulgaris* reads and obtained a group of contig measures: the group of Nx50, percentage of the genome covered with a contigs and a number of misassemblies, all the ones generated with Quast^29^. The results are presented in Fig. 4.

In Supplementary material, we present the additional assembly results, the results obtained with an assembler Velvet ^30^ and the results for *P. syringae*. We also presents results of reads mapping experiments with BWA-MEM and the ones showing reads simulated with ART^31^ correction efficacy.

In most cases, the best results are obtained for reads corrected with Blue and RECKONER 2. However, those ones (together with Lighter) yield the highest number of missassemblies. It is visible, that number of them is the best for RACER, Fiona, and Blue, which in all the other cases turned out the worst ones. SAMDUDE results were very similar to the uncorrected reads and Lighter, in most cases, gives a slight improvement. Therefore, the results are a compromise between obtaining longer contigs and the better ones.

The correctors that utilize quality scores are RECKONER, BLESS, Lighter, CARE, and SAMDUDE. The other do not or we have no information about it. The experiments showed, that NovaSeq quality characteristics may impact BLESS and CARE, as they achieved poor results. Both versions of RECKONER are not affected, whereas for Lighter and SAMDUDE it is not obvious.

**Table 2:** Correction time and memory requirements for *H. sapiens*.

	Seq. depth	Algorithm								
		RECKONER	RECKONER 1	Musket	RACER	BLESS	Blue	Lighter	BFC	CARE
Time [h]	$15\times$	0.79	0.92	1.65	2.22	1.55	–	1.64	0.84	4.78
	$30\times$	1.34	2.37	3.25	4.17	4.03	–	3.22	1.62	11.49
	$45\times$	1.82	2.74	4.87	6.24	5.36	69.93	4.54	2.49	–
	$60\times$	2.39	4.27	8.33	9.55	7.46	–	6.47	3.29	–
RAM [GiB]	$15\times$	24.91	22.13	5.4	154.46	7.45	–	13	53.45	242.31
	$30\times$	33.18	8.88	9.88	172.28	7.44	–	13	53.44	248.48
	$45\times$	36.49	10.6	14.28	189.84	7.45	228.01	13	53.45	–
	$60\times$	36.45	10.72	41.81	207.32	7.44	–	13	53.44	–

Table 2 shows time and memory consumption of different correctors for human read datasets processing, for algorithms being able to correct at least one set. The fastest tools are RECKONER 2 and, slightly slower, BFC. They are able to correct high-depth reads in less than 2.5 and 3.5 h, respectively. The other ones require at least 6.5 hours (Lighter) up to 9.5 (RACER), excluding CARE, which was not able to correct some cases, but it seems, that its work may need significantly more time. The results show, that all the algorithms scale rather well, hence the time of correction is approximately linear in a function of the sequencing depth.

Memory consumption varies extremely. The lowest requirements were noticed for Bloom-filter-based BLESS and Lighter, which are greatly memory-frugal, and insensitive for the sequencing depth. Musket, the third one based on Bloom filter, does not seem to have a similar behavior. BFC and RECKONER 2 are also more-or-less RAM-constant and their needs are rather moderate. The fluctuations noted for RECKONER 1 are caused by different requirements of *k*-mer length for different depths. RACER and CARE need a huge amount of memory. In fact, CARE has a parameter limiting it (in the experiments, it was set to 250G), but reducing it may be problematic, as it failed for $45\times$ and $60\times$ depth due to a memory lack.

**Table 3:** Correctors ranks according to various criteria.

Results	RECK. 2	RECK. 1	Musket	RACER	BLESS	Fiona	Blue	Lighter	BFC	Karect	SAMDUDE	CARE
Memory	3	5	2	7	4	10	9	1	6	12	11	8
Time	3	2	6	7	4	8	10	5	1	11	12	9
NGA50 (Minia)	2	3	10	8	12	8	1	5	4	8	11	6
NGA50 (Velvet)	2	3	5	7.5	10	11	1	5	5	7.5	12	9
Mapped reads	8	6	5	3	12	1.5	4	9	7	1.5	11	10
Mapped once	3	10	7.5	11	12	1	5	9	5	7.5	2	5
Gain (sim.)	3	5	7	6	9	2	8	10	4	1	12	11
Gain-nuc (sim.)	3	5	6	10	7.5	1	9	7.5	4	2	12	11
*H. sapiens* VC	4	6	2	8	5	11	9	1	3	11	11	7
*A. thaliana* VC	3.5	3.5	10	11	1	12	6	8	2	9	5	7
Sum	34.5	48.5	60.5	78.5	76.5	65.5	62	60.5	41	70.5	99	83

Due to a significant number of evaluating criteria, frequently non-consistent, we calculated a synthetic ranking. Table 3 shows corrector ranks obtained for various criteria, similarly to Ref.^17^ (smaller values are better). For all the experiment types, we took two values. The memory and time indicators are results of all the shown experiments on real reads; $\text {gain}$ is a measure for simulated reads, defined as shown in Supplementary material; $\text {gain-nuc}$ is an analogous measure, but defined for single nucleotides; VC comparison was performed by utilizing both SNP and indels results for all the sequencing depths and calling pipelines. One has to have in mind, that the rank gives just a general outlook, and a decision on an algorithm choice has to meet also specific criteria, e.g. RECKONER 2 is the fourth for human VC, but it may be not appropriate for VC aimed at indels. On the other hand, it is behind Musket and Lighter, which are slightly overstated due to the effect of actual leak of changes introduced to the reads.

## Conclusions

The aims of this study were: to assay impact of the Illumina reads correction on a variant calling results, verify the NovaSeq reads correction viability, and boosting and optimizing one of the correctors, i.e., RECKONER.

The experiments showed, that correction evaluation with the VC quality is a hard task, drawing not a clear conclusions. It suffers from the lack of the ground truth data, limiting the range of possible explored cases. Utilization of the *A. thaliana* data is not straightforward, due to an imperfection of the available data. Moreover, the technical difficulties are also the case. Strelka variant caller generates non-typical variants quality values. Correction of human reads, then mapping them, VC and results evaluation are computationally difficult tasks, requiring a huge amount of time. The problems explain low popularity of such an analysis method in the literature. The results are hard to be compared with another works, as they have a limited scope and there are not reviews assays or they are limited to a single corrector. Despite that, the case should be analysed deeper to provide a template to further correctors assays.

The results of de novo assembly showed, that some of correctors are resistant to different characteristics of the NovaSeq reads. The problem may be with algorithms utilizing the values, as in that group only RECKONER allowed to obtain good results.

The experiments proved, that there still exists an opened subject of a *k*-mer length tuning analysis. Most of the correctors need a defined value, whilst the algorithms guidelines, if at least given, are not precise and easy to apply. In this work, we were able to choose the best values experimentally, at the expense of the high-time-consumpting experiments. In the practical applications, it is rather done by an arbitrary choice of some value, with no knowledge about its feasibility.

In the work we shown, that a new version of corrector RECKONER proves itself as being better than the predecessor, typically giving better results in less time, and keeping moderate memory requirements. The new version is a gap-filling of a poor represented short indel errors correction field. In general, it appears to be the best solution, when taking into account all the analyzed fields of utilization.

## Methods

### RECKONER 2 workflow

With aim to achieve better speed and quality results we boosted RECKONER algorithm with a set of novelties. RECKONER belongs to a group of *k*-spectrum-based algorithms by a taxonomy of Yang et al.^11^ and its high-level working scheme includes:

(*i*): verifying input files formats and determining the parameters,
(*ii*): counting of *k*-mers and, optionally, $k'$-mers,
(*iii*): analysing of *k*-mer *counts* (numbers of their occurrences) histogram aiming to detect the threshold, which distinguishes *k*-mers with high probability originating from erroneous reads,
(*iv*): removing of likely erroneous *k*-mers; remaining *k*-mers are called *trusted*, and
(*v*): independent correcting of the reads by modifying them to achieve sequences containing just trusted *k*-mers.

The fundamental (*v*) stage consists of two main steps performed for every read: detection of read *regions* (subsequences) containing errors, what is done by querying a *k*-mers database with aim to detect the missing (untrusted) ones, and correction of the appointed regions. The latter is performed with a backtracking algorithm changing the consecutive region symbols to achieve such *k*-length read subsequences, which will constitute trusted *k*-mers.

In the newest version, the backtracking algorithm utilizes the *correction sequence* containing the changes, which should be introduced to the region in the currently checked solution, what could be also interpreted as a state of regions correction process. The sequence $(\mathfrak {s}_i)$ contains tuples:

1 $$\begin{aligned} \mathfrak {s}_i = (n, c, \text {ins}, \text {del}, \text {exp}\_\text {ind}), \end{aligned}$$

where: *n* is a count of the *k*-mer present in the read, the last (or the first, in a case of regions situated at the $5'$ end of the read) symbol of the *k*-mer that is going to be changed according to guidelines in $\mathfrak {s}_i$, $c \in \{\textsf {A}, \textsf {C}, \textsf {G}, \textsf {T}, \varnothing \}$ is the new symbol of the *k*-mer (in a case of correcting a substitution or deletion error) or indicator $\varnothing$ of leaving the symbol untouched or removed. Flags $\text {ins}$ and $\text {del}$ are logical values indicating, that correction should depend on, respectively, removing one or attaching a new symbol (i.e., correcting the indel error). A flag $\text {exp}\_\text {ind}$ indicates, that while performing a backtracking, another possibility relying on checking correction of the indel should be considered—it could cause setting $\text {ins}$ or $\text {del}$ flag in the future. Obviously, the following relation is true: $\text {ins}\, \wedge \lnot \text {del}\, \wedge c=\varnothing \vee \lnot \text {ins}\, \wedge c \ne \varnothing$.

The abovementioned representation of the algorithm state allows to correct single indels present in the read, which is being done with the following method. The region is processed symbol-by-symbol by changing them (or leaving the original ones) by correcting simple substitutions to achieve the trailing *k*-mer to be trusted (building a solution—traversing towards leaves in a search tree of the backtracking algorithm). Decision about a correction of every symbol is stored in the stack. If the end of the region is reached, the stack is stored as a correction path, what is a potential solution. However, presence of (nearly) adjacent of many changes in the stack (at least 3 changes in the sequence of 6 positions) suggests, that an indel error could occur. In such a case, the first symbol of the group of changes obtains a corresponding value $\text {exp}\_\text {ind} = \text {true}$ and while backtracking the algorithm (returning towards the tree root) such an indicator initiates consideration of the new group of changes in the region: removing the symbol or inserting one of the set $\{\textsf {A}, \textsf {C}, \textsf {G}, \textsf {T}\}$, which is done by setting a proper flag in the stack. It is a trial of correction of an insertion or deletion error, respectively. The changes form new branches in the backtracking tree.

Multiple paths generated with the described algorithm are rated according to the equation (for the correction towards $3'$ end):

2 $$\begin{aligned} r(a, b, \mathscr {K})=\frac{\sum \limits _{\kappa \in \mathscr {K}}^{}\text {weight}(\kappa )n(\kappa )}{\sum \limits _{\kappa \in \mathscr {K}}^{}\text {weight}(\kappa )}\left( \prod \limits _{i=a+k-1}^{b+k-1}\text {prob}(i)\right) \theta ^{n_\text {ind}}, \end{aligned}$$

The parameters *a* and *b* are indices of the first and the last *k*-mer of the erroneous region (the original ones, i.e., not modified by the indel correction, yet), $\theta = 0.001$ is an approximate probability of the indel error for some position in Illumina reads, $n_\text {ind}$ is a number of indels corrections, $\kappa$ is a *k*-mer with sequence after corrections applying, $\mathscr {K}$ is a set of all *k*-mers, which the last symbols belong to the region after applying the modifications, $n(\kappa )$ is a $\kappa$ count (stored in the stack). The functions $\text {prob}$ and $\text {weight}$ are defined by Eqs. 3 and 4:

3 $$\begin{aligned} \text {prob}(i)= {\left\{ \begin{array}{ll} 1, \text { when symbol on } i\text {-th position was modified,}\\ p[i]\text { otherwise,} \end{array}\right. } \end{aligned}$$

where *p*[*i*] is a probability, that the corresponding symbol is erroneous, what is delivered with the input files,

4 $$\begin{aligned} \text {weight}(\kappa )= {\left\{ \begin{array}{ll} 0.5\text {, when }\kappa \text { is an extending }k\text {-mer,}\\ 1\text { otherwise,} \end{array}\right. } \end{aligned}$$

where an extending *k*-mer is the best one, which arose by attaching a single symbol at the end of the read or region (details described in Ref.^32^ and in a Supplementary material of Ref.^17^).

As changed symbol error probabilities and $\theta$ values are multiplied, the rates should be higher for paths with lower number of changes. It is typical for *k*-spectrum-based algorithms, that they prefer conservative corrections, as it reduces a chance, that correction would cause altering the region to the form representing a completely different region of the genome.

Another novel extension of RECKONER 2 is a capability to verify corrections by utilization of long *k*-mers ($k'$-mers, by default $k'=1.5k$). It includes another step of correction, where $k'$-mers are extracted from the corrected read and queried in a second database of the $k'$-mers. A necessary condition for the read sequence acceptance is a presence of all of them in the database, but the count is not taken into account, i.e., just a single occurrence of the $k'$-mers in the input reads is sufficient. If the condition is not satisfied, another path is chosen, from the sets of regions correction paths, and applied to the read and the verification is repeated. The $k'$-mers verification is especially designed to facilitate de novo assembly, hence it is not enabled by default and can be used by a user when necessary.

### Correction example

**Figure 5:**
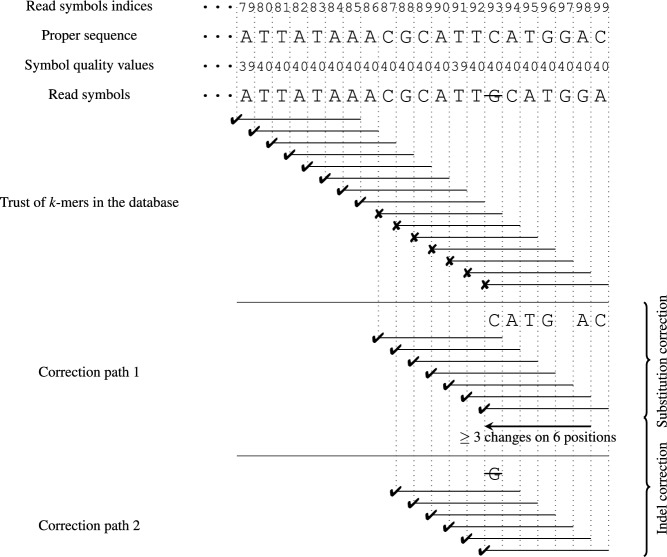
Indel error detection and correction, $k=7$.

Figure 5 shows the idea of indels correction. Let us suppose, that a read contains an inserted erroneous symbol G on its 93 position. *k*-spectrum-based algorithms would extract *k*-mers from the read and verify their presence in the database, what in a typical case would cause an absence of the *k*-mers covering the read fragment containing the problematic symbol. It is a basic premise, that symbol on the position 93 is erroneous, however, with no direct information about an error type.

Such a situation would be corrected by RECKONER 2 by changing consecutive read symbols, starting with 93 and verifying, if the change (or leaving the original symbol) causes the trailing *k*-mer (e.g., for symbol in position 93 it will be a 7-mer starting on position 87) become trusted. If such a possibility is found, the next symbol is changed and the next *k*-mer is verified. The changes aim to achieve a proper, but not known in advance, sequence. Finally, a (typically small) set of changes will be devised, which is an excellent premise to correct possible substitution errors. However, our case includes just an insertion error.

It is possible, that the above algorithm would generate a sequence of changes denoted in the figure as Correction path 1. Of course, as in a moment of choosing a symbol many possibilities may be found, number of achieved paths could be bigger than one. However, the path contains many changes, which would be caused by a highly-erroneous region of the read or a presence of the indel. Both the cases will be verified. The path is a stored copy of stack of size 7, containing respectively values of *c*: $\textsf {C}, \textsf {A}, \textsf {T}, \textsf {G}, \varnothing , \textsf {A}, \textsf {C}$, all indicators set to $\text {false}$ and stored counts of the *k*-mers (not shown).

Awhile building a path, the algorithm observes a number of changes stored in the stack. If it exceeds the threshold of 3 changes in a sequence of 6, the symbol on the beginning of the sequence is marked by setting $\text {exp}\_\text {ind} = \text {true}$ in the stack, which would happen just when correcting position 98 for position 93. After that, a stack element is changed to $\mathfrak {s}_0 = (n, \textsf {C}, \text {false}, \text {false}, \text {true})$ (with some *n* value). Next, the correction is continued normally.

After backtracking the algorithm, it is checked if the successive (but in reverse order) positions are marked by $\text {exp}\_\text {ind} = \text {true}$. It is the case for position 93, and all of the five indel changes (removing a symbol or inserting all ones of four possible missing symbols) are verified for that position. After finding a trusted *k*-mer, the typical substitution algorithm is continued. In the exemplary correction, the indel correction would cause removing of the symbol G, and, finally, all of the following *k*-mers would become trusted with no another changes, which constitutes Correction path 2. Both of the paths have their own rates, and the path with the highest one is eventually applied to the read. The stack contains the following values: $\mathfrak {s}_0 = (n, \varnothing , \text {true}, \text {false}, \text {false})$, for some count *n* of the first *k*-mer, and the other elements would contain just some *k*-mers counts, indicators of value $\text {false}$ and $c = \varnothing$.

It is necessary to mention, that in the example it is possible to correct the insertion without adapting the sophisticated indel correction by applying Correction path 1. The case is caused by the presence of the insertion close to the end of the read, when indeed indel correction may be achieved with multiple substitution fixes. However, when the indel is situated deeply inside the read, it would not be possible. Substitution error corrections would be performed, but surrounding them by a regions of the read classified by the algorithm as devoid of errors cause problems with achieving situation, when all the *k*-mers are trusted. Moreover, leaving a substitution-only algorithm on the edges of the read would be also a problem, as obtained paths can contain many of corrections, which would achieve an extremely small rates and problems with choose a proper path.

### Experiments details

All sequencing reads were paired. The desired sequencing depths are obtained as follows:

- for human reads we concatenated the appropriate pair files, respectively: single ERR174324 for $15\times$, ERR174324 and ERR174325 for $30\times$ and so forth,
- for *A. thaliana* (depths of $10\times$, $20\times$, ..., $100\times$) and *C. vulgaris* ($60\times )$ we shuffled the read pairs and extracted a number of them from the beginning of the shuffling result.

To call variants we passed both raw and corrected reads with different algorithms to BWA-MEM^21^ with aim to map them to the reference genome. Then the mappings were passed to Strelka^22^. The exception was SAMDUDE, which requires as an input the mapped reads, hence in that case we mapped the raw reads, passed the mappings to SAMDUDE, and its results (also mapped) passed to Strelka. The outgoing variants sets were evaluated with hap.py by comparing them with the ground truth sets. In a case of human experiments, confident call regions set (BED files) was also utilized. The results of hap.py are, i.a., statistical measures of recall and precision. To synthesize them, we evaluated F1-score as their harmonic mean. We selected the measures for variants, that not necessarily passed filters (marked as ALL). SNP in hap.py terminology means a single- or multiple nucleotide polymorphism, while indel means a simple indel or complex variant.

In a case of de novo assembly, the corrected and raw reads were independently passed to Minia. For SAMDUDE reads were mapped to the genome with BWA-MEM, then corrected, extracted from the resulting SAM file and passed to the assembler.

To determine *k*-mer length, which is a parameter of most of correctors, we run them with a range of *k* values (in a case of de novo assembly values were consecutive, in a case of VC just odd values) and the best one was chosen. The criterion of rating was a harmonic mean of F1-score for SNPs and indels (for VC) and optimizing all the statistics Nx50 and Lx50 (for assembly). Exact values are available in the Supplementary material.

### Supplementary Information

Supplementary Information.

## Data Availability

All of the sources of the data utilized in the experiments are stated in the Supplementary material.

## Code availability

The code of the described RECKONER 2 (version 2.1) is available at https://github.com/refresh-bio/RECKONER.
