# Supplementary material for: Illumina reads correction: evaluation and improvements
Source: Sci Rep. 2024 Jan 26;14:2232. doi: 10.1038/s41598-024-52386-9 (PMC11222498; doi:10.1038/s41598-024-52386-9)
Supplement: Supplementary file 1 — Supplementary Information. [file 41598_2024_52386_MOESM1_ESM.pdf]

Illumina reads correction — evaluation and improvements

Maciej Długosz Sebastian Deorowicz

Contents

1 Experimental results **2**
1.1 Human variant calling . . . 2
1.1.1 Variant calling results — genotype concordance . . . 2
1.1.2 Variant calling results — homo- vs heterozygous variants . . . 2
1.2 *A. thaliana* variant calling . . . 2
1.3 *De novo* assembly . . . 2
1.4 Reads mapping . . . 2
1.5 Simulated reads . . . 3

2 Cases studies **19**
2.1 NovaSeq reads characteristics impact . . . 19
2.2 Correction impact on variant calling . . . 19

3 Data sources **26**

4 Experiments details **28**
4.1 Algorithms versions . . . 28
4.2 Running the correction algorithms . . . 28
4.3 Running the other algorithms and pipelines . . . 29
4.4 Experiments environment . . . 31
4.5 Experiments failures . . . 31

5 Exact algorithms parameters **34**

1 Experimental results

1.1 Human variant calling

Figure 1 shows results for *H. sapiens* VC with Strelka, evaluated with hap.py (similar to the main maper). Missing bars correspond to experiments, when the correction failed. Due to low best (i.e. maximizing geometric mean of F-1 scores for SNPs and indels) k values for Lighter and Musket we performed the experiments also for the maximal odd values of that parameter ($k = 31$ and $k = 27$, respectively — for higher values the results do not change). The results are shown on Figure 2.

Figure 3 shows results for *H. sapiens* VC with DeepVariant, evaluated with hap.py. In the experiments, we used the same reads like for experiments with Strelka and hap.py.

Results are different from the ones obtained with Strelka. Especially, there are observable poor outcomes of RECKONER and BFC for 15× set. In general, results similar to the uncorrected reads are obtained just for practically non-correcting Lighter and Musket and CARE (which was not able to perfrom correction for 45× and 60×). For indels, CARE and — for at least 30× depth — BFC achieve similar results to the raw reads, but it may be due to none of them is designed to correct indels.

Figure 4 shows results for *H. sapiens* VC with Strelka, evaluated with Syndip. In the experiments, we used the reads from a ERR1341796 dataset. To generate different sequencing depths we shuffled the read pairs and extracted a number of them from the beginning of the shuffling result, similarly like for *A. thaliana*. The whole input dataset sequencing depth is 55×, hence we chose it instead of 60×.

1.1.1 Variant calling results — genotype concordance

Figure 5 shows genotype concordance sensitivity and precision results for *H. sapiens* VC with Strelka. Measures were computed with GATK.

1.1.2 Variant calling results — homo- vs heterozygous variants

Figure 6 shows F1-score results *H. sapiens* VC with Strelka, evaluated with hap.py (similar as shown in Figure 1) separately for homorozygous and heterozygous variants. We calculated the values by splitting both GT and obtained variants to two sets: containing 1/1 (or 1|1) and 0/1 (or 0|1) GT fields, respectively. Then we evaluated the sets with hap.py.

Unfortunately, the only algorithm with an option for homo- and heterozygous data differentiate — Karect — was not able to correct the reads. In a case of another algorithms clearly visible is a huge improvement of heterozygous SNPs for 15× set. It is a desirable behavior, as one can expect, that the non-heterozygous-aware algorithms may degrade these case. For higher depths the phenomenon gradually disappears, and finally results for the homo- and heterozygous variants become similar, expect for RECKONER 2, which deal better with heterozygous indels, and Blue, which generally seems to be strongly adapted to homozygous variants.

1.2 *A. thaliana* variant calling

Figure 7 shows additional results for *A. thaliana* VC with Strelka, evaluated with hap.py.

Figure 8 shows results for *A. thaliana* VC with DeepVariant, evaluated with hap.py. In the experiments, we used the same reads as for experiments with Strelka and hap.py.

1.3 *De novo* assembly

Figure 9 shows additional *de novo* assembly measures for *C. vulgaris*. Figure 10 shows *de novo* assembly measures for *C. vulgaris*, assembled with Velvet.

Figure 11 shows *de novo* assembly measures of long MiSeq reads for *P. syringae*. Figure 12 shows *de novo* assembly measures for *P. syringae*, assembled with Velvet.

1.4 Reads mapping

Figure 13 shows reads mapping characteristics for *C. vulgaris*. Measures once and multiple denote a fraction of the reads mapped once and multiple times to the reference genome, respectively. Measures ins and del denote a fraction of reads affected by insertion or deletion, respectively. A measure frac(d) denotes a fraction of reads differing from the genome with an edit distance d.

1.5 Simulated reads

Figure 15 shows results of correction reads generated *in silico*. The measures are defined as follows: gain = $\frac{|TP|-|FP|}{|TP|+|FN|}$, sensitivity = $\frac{|TP|}{|TP|+|FN|}$, precision = $\frac{|TP|}{|TP|+|FP|}$, where $|TP|$ is a number of the reads perfectly corrected, $|FP|$ is a number of the reads destroyed by a corrector, and $|FN|$ is a number of the reads not corrected or not fully corrected. Those measures may be counted in term of corrected nucleotides rather than reads. The notation LxDy means the read length equal to x bp and the sequencing depth equal to y.

The simulation was performed with ART. As quality profiles we utilized the first reads of pairs from the sets DRR031158 (for length 100 bp) and SRR1802178 (for 150 bp). As k-mer lengths we used the best value for L100D20 cases.

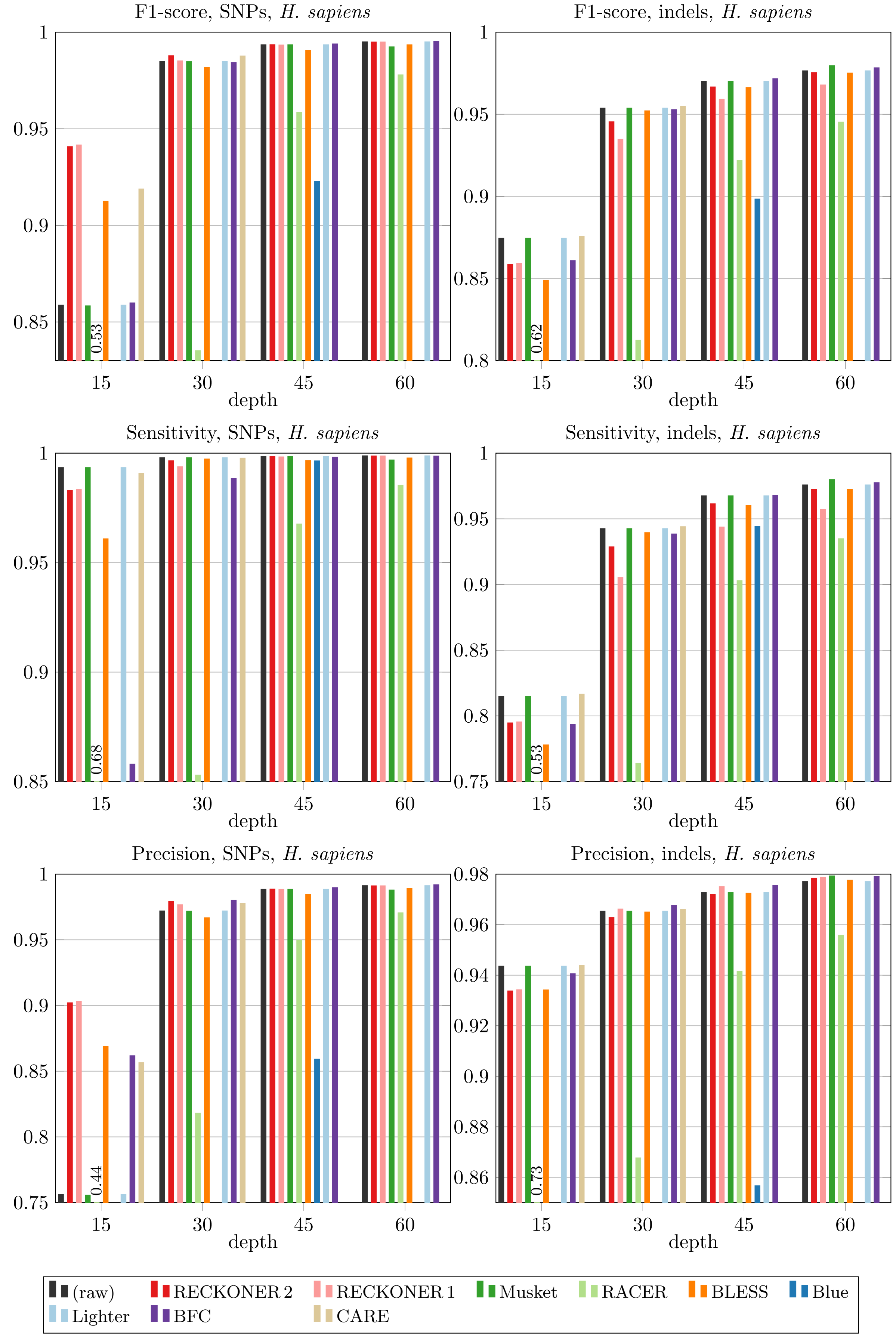

Figure 1: Results for *H. sapiens* VC — Strelka and hap.py

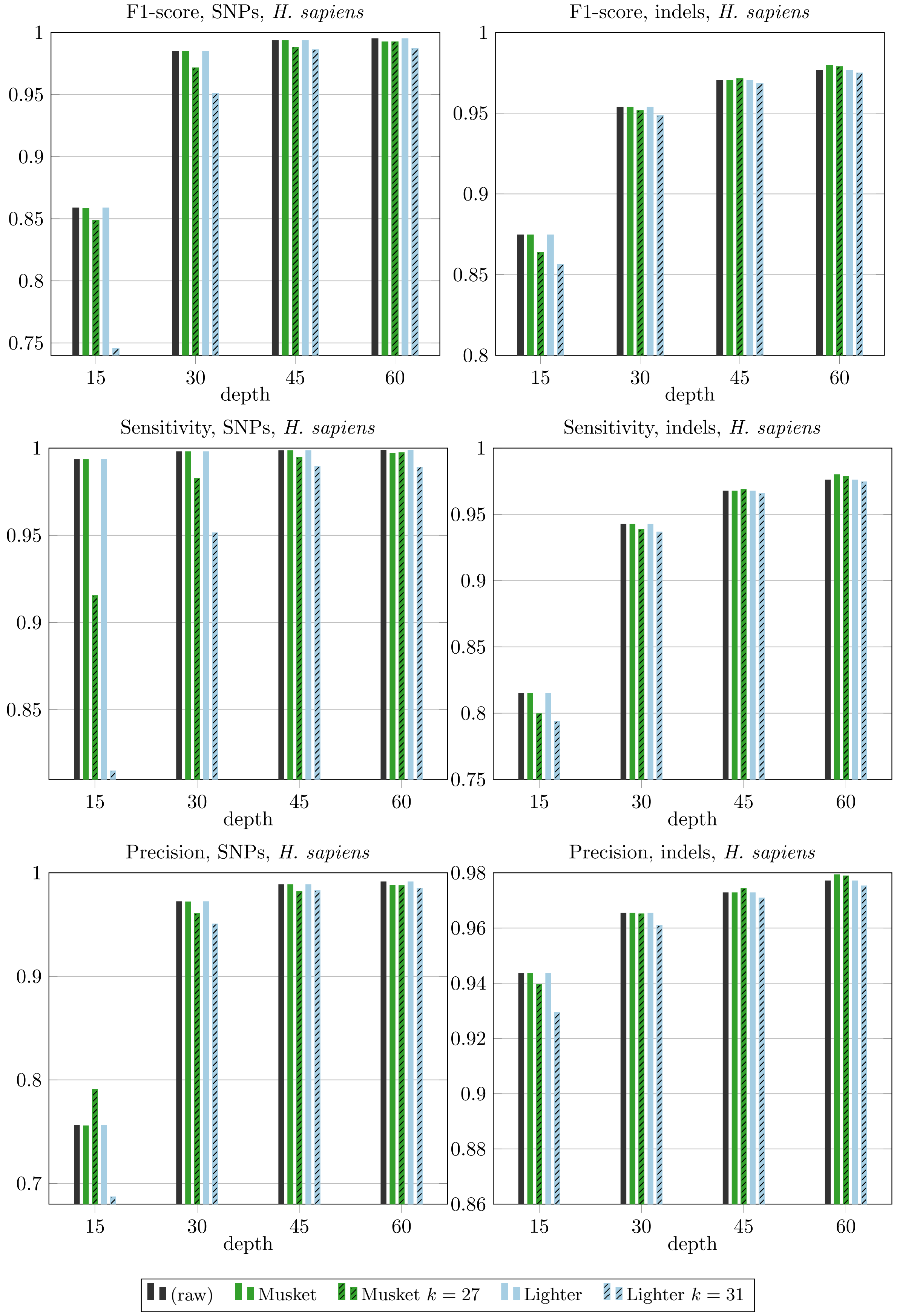

Figure 2: Results for *H. sapiens* VC — Strelka and hap.py, correction with Lighter and Musket

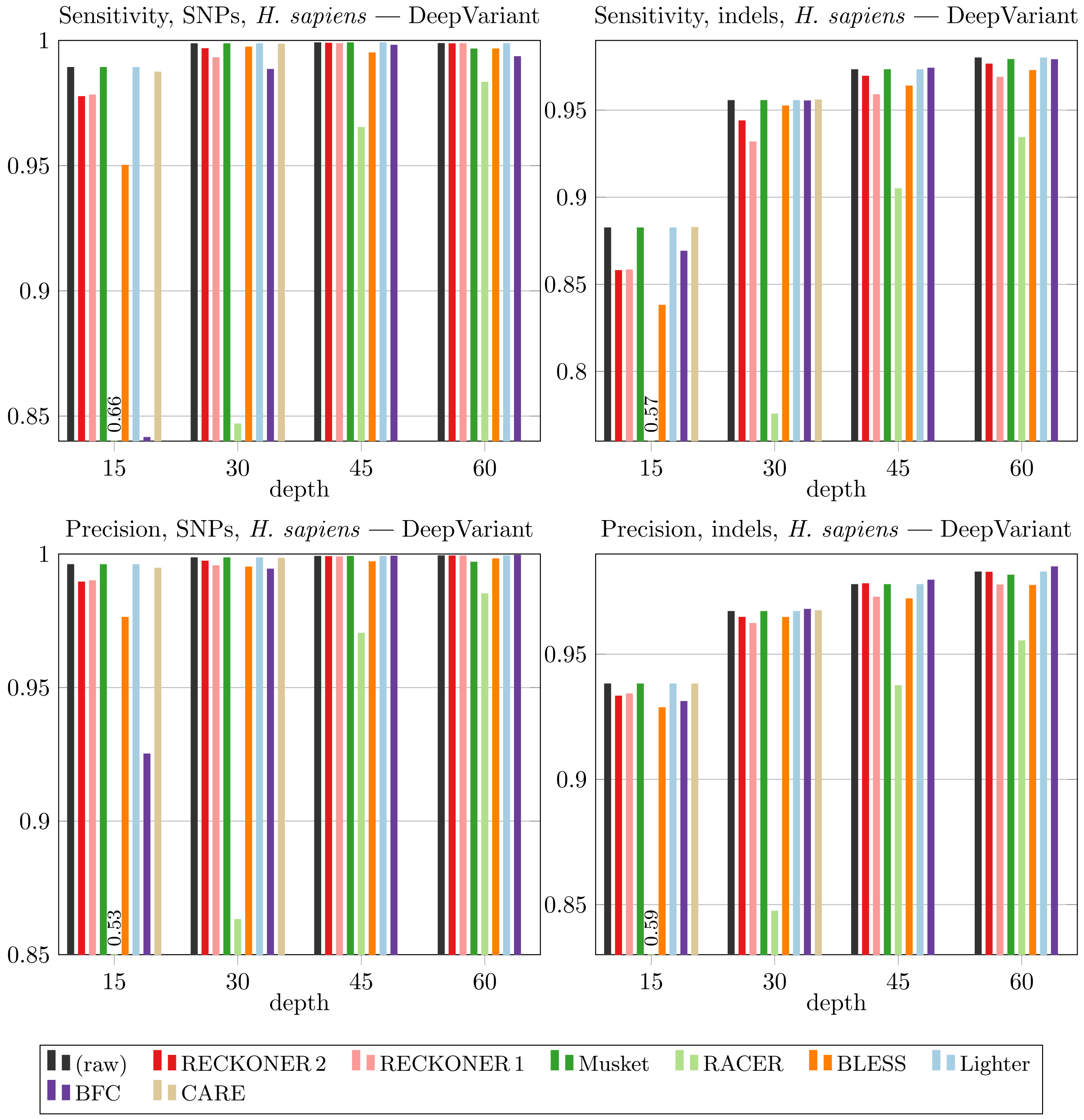

Figure 3: Sensitivity and precision results for *H. sapiens* VC — DeepVariant and hap.py

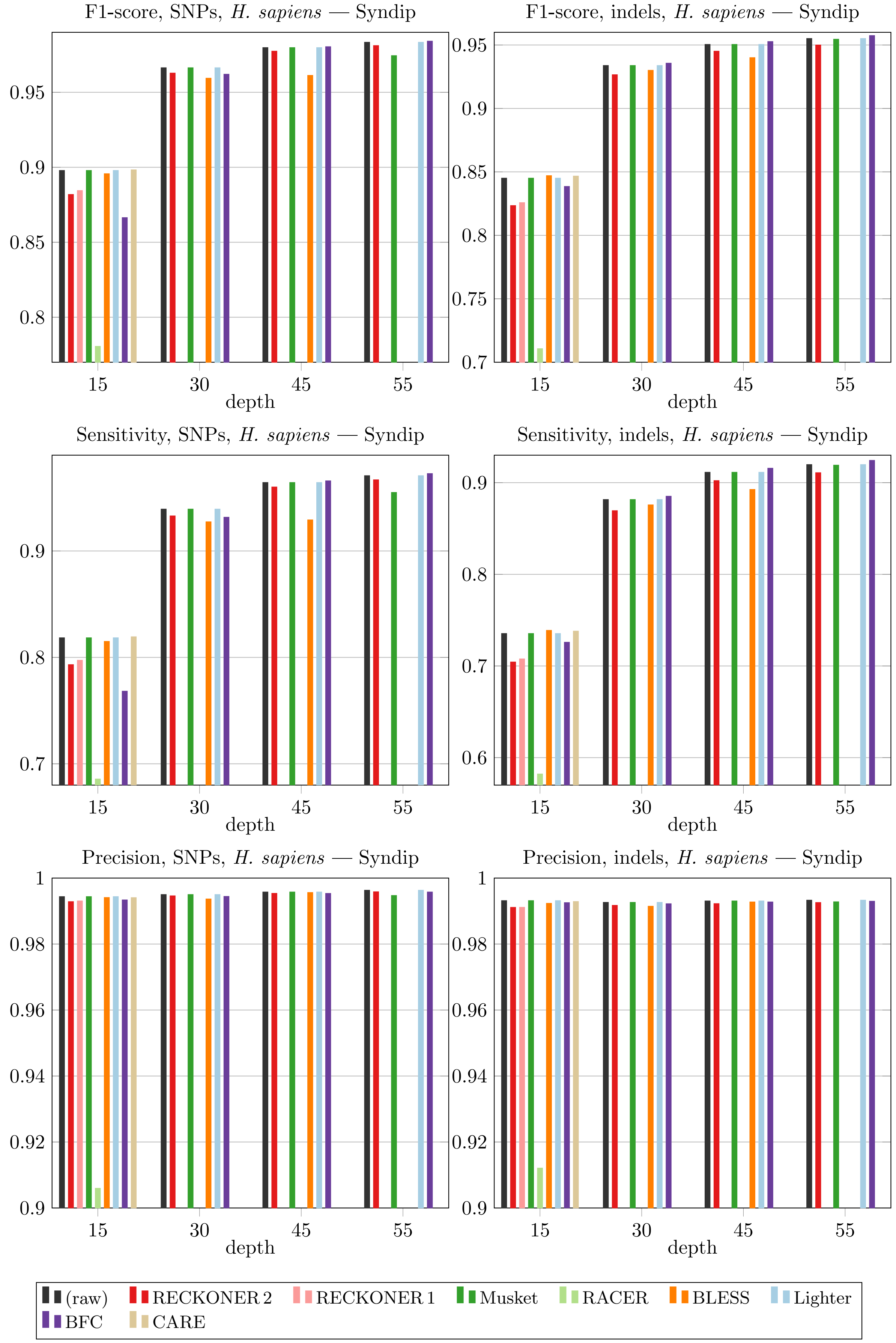

Figure 4: Results for *H. sapiens* VC — Strelka and Syndip

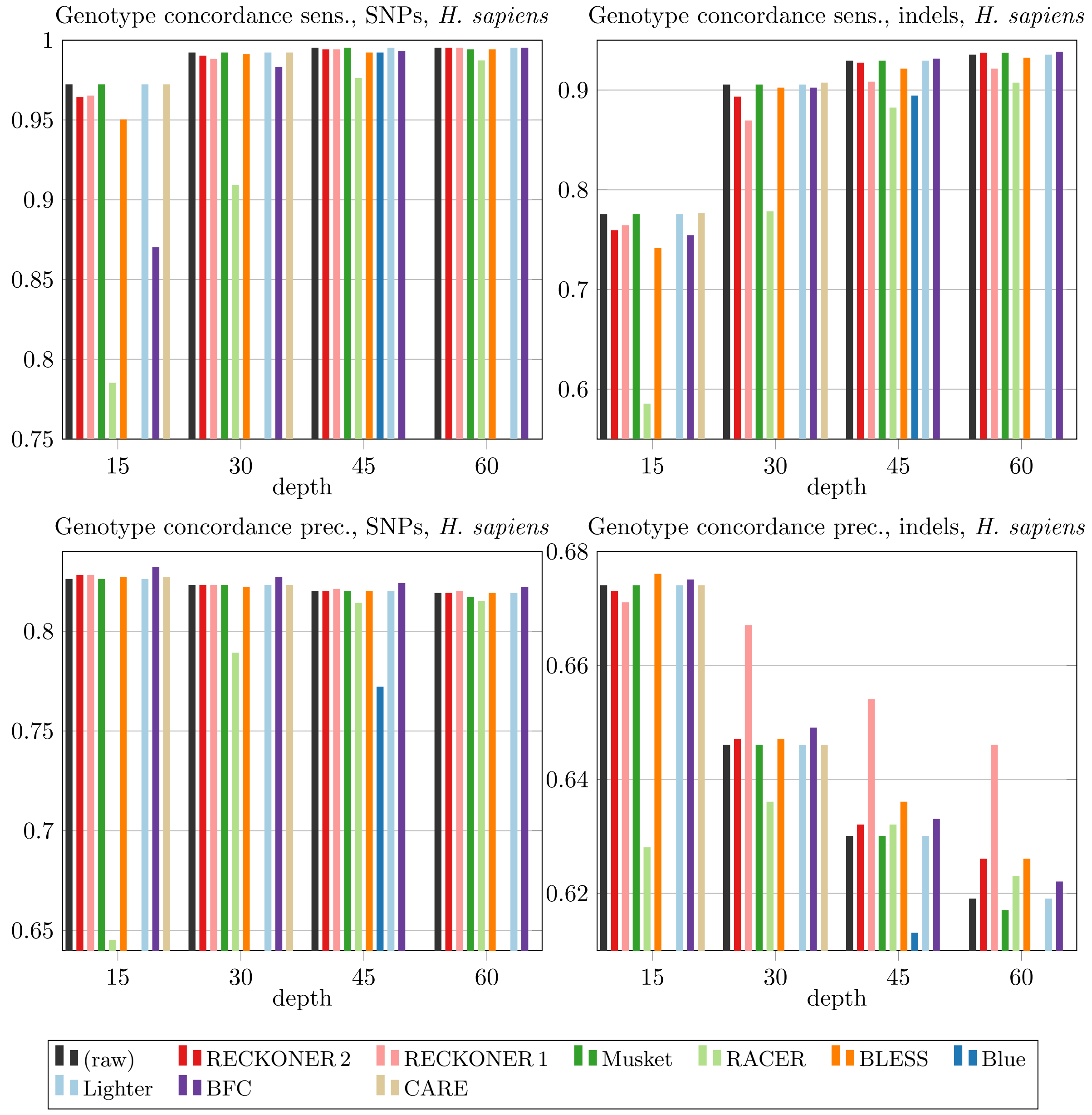

Figure 5: Genotype concordance sensitivity and precision results for *H. sapiens* VC and hap.py

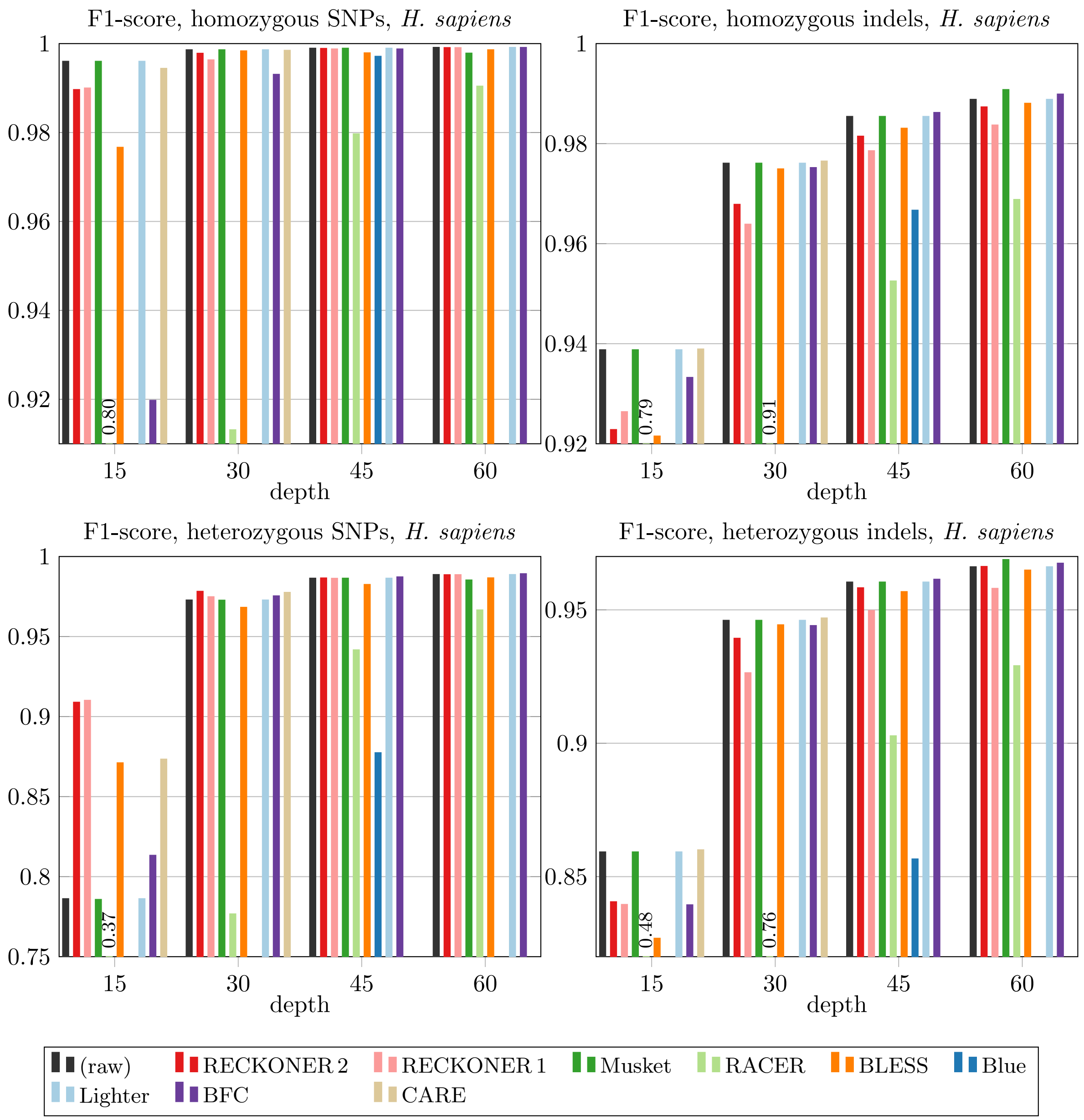

Figure 6: F1-score results for *H. sapiens* VC, separately for homo- and heterozygous variants — Strelka and hap.py

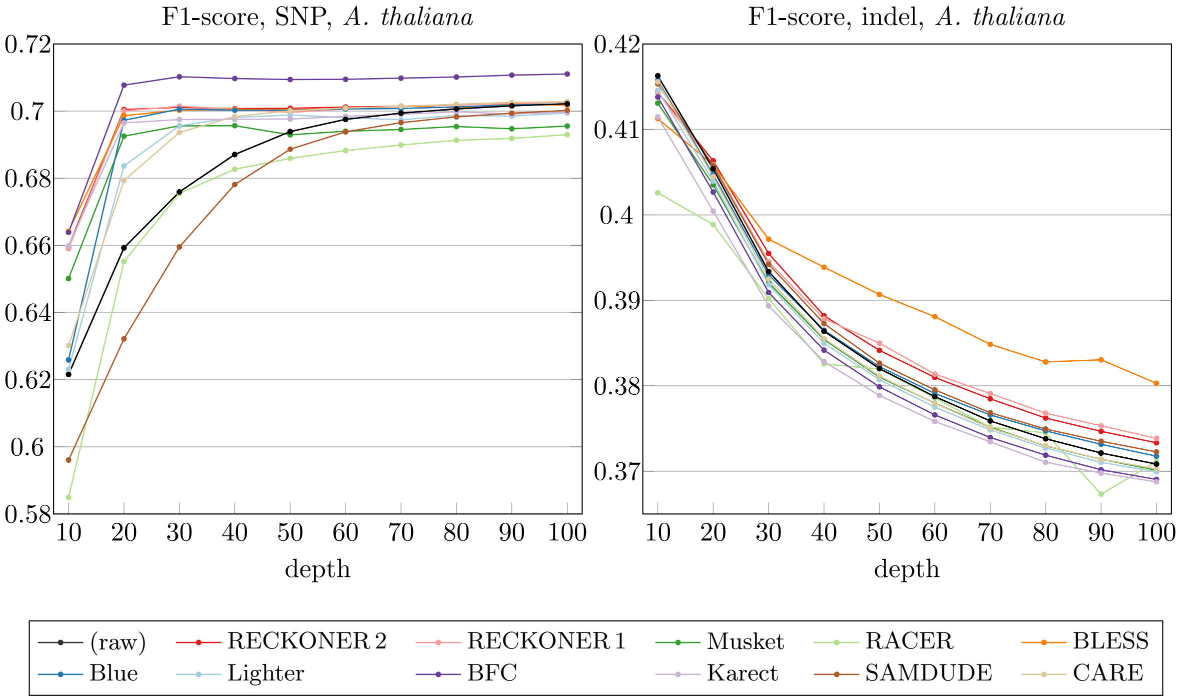

Figure 7: F1-score for *A. thaliana* VC — Strelka and hap.py

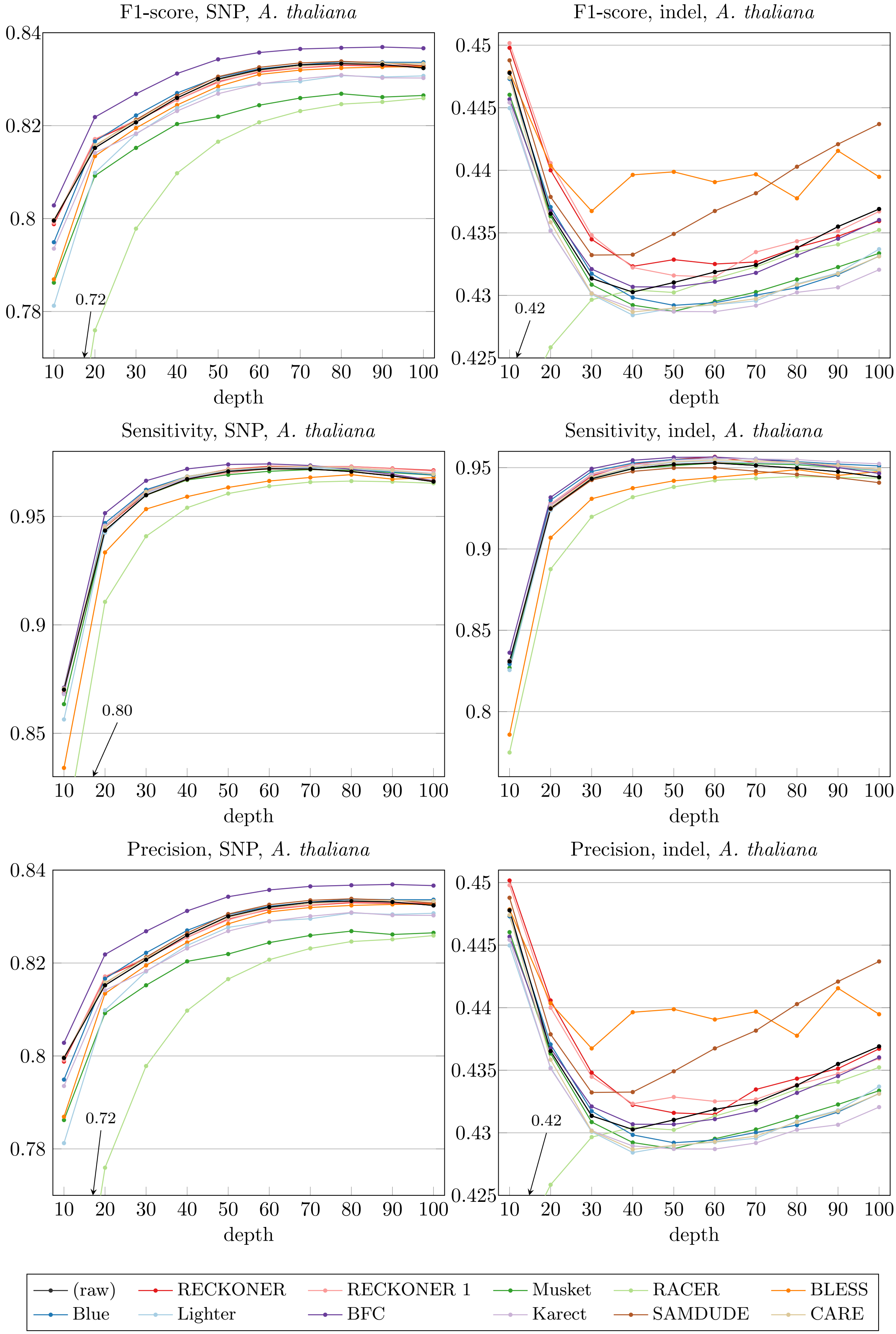

Figure 8: Results for *A. thaliana* VC — DeepVariant and hap.py

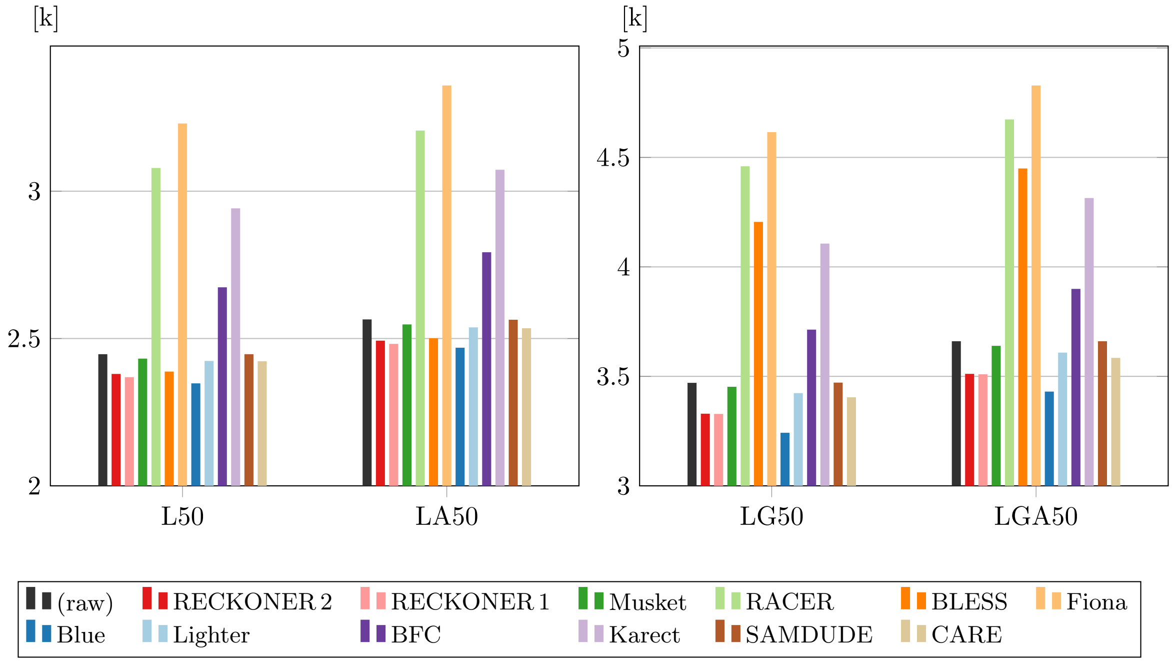

Figure 9: Assembly quality of NovaSeq *C. vulgaris* reads — Minia

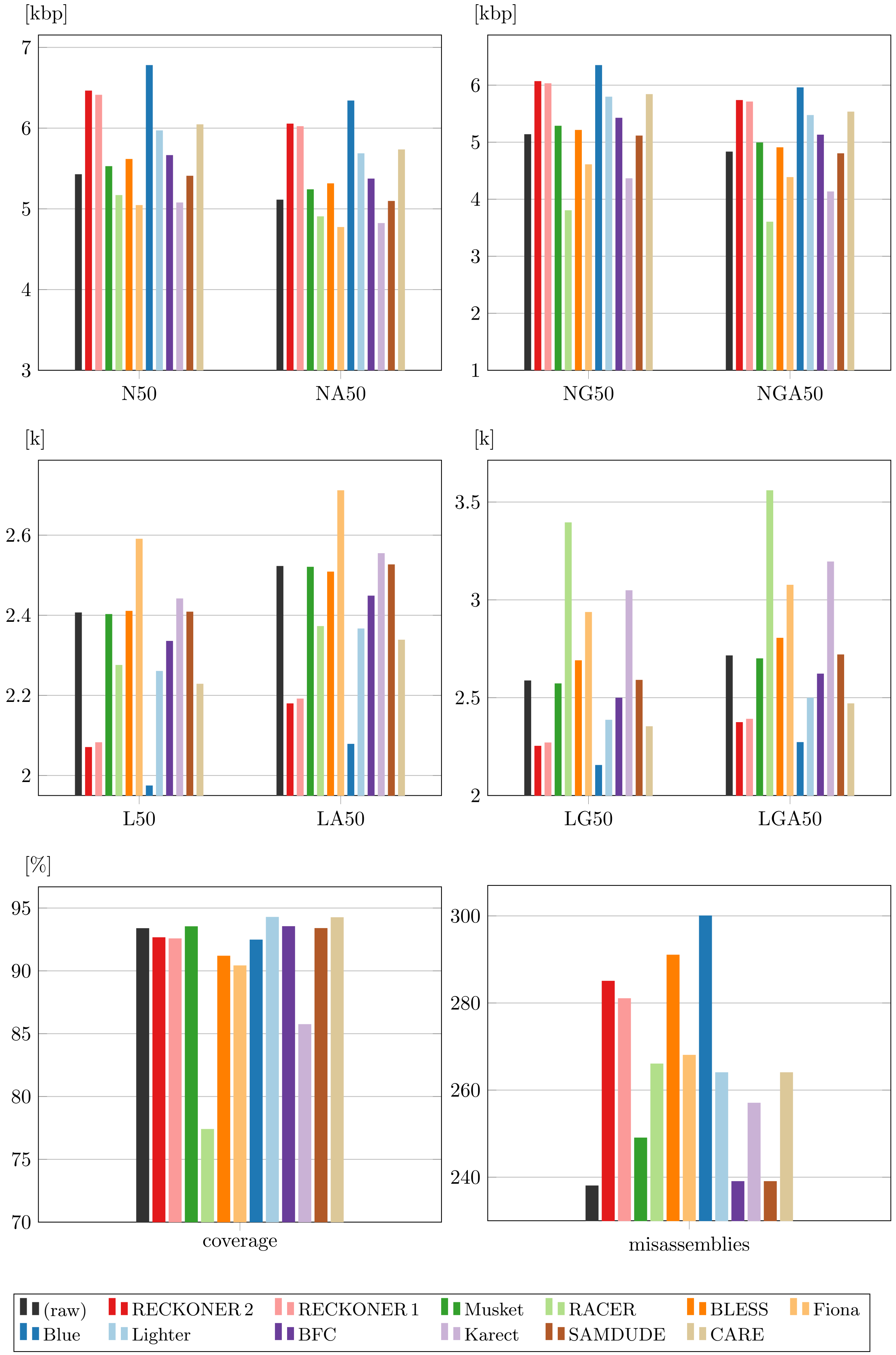

Figure 10: Assembly quality of NovaSeq *C. vulgaris* reads — Velvet

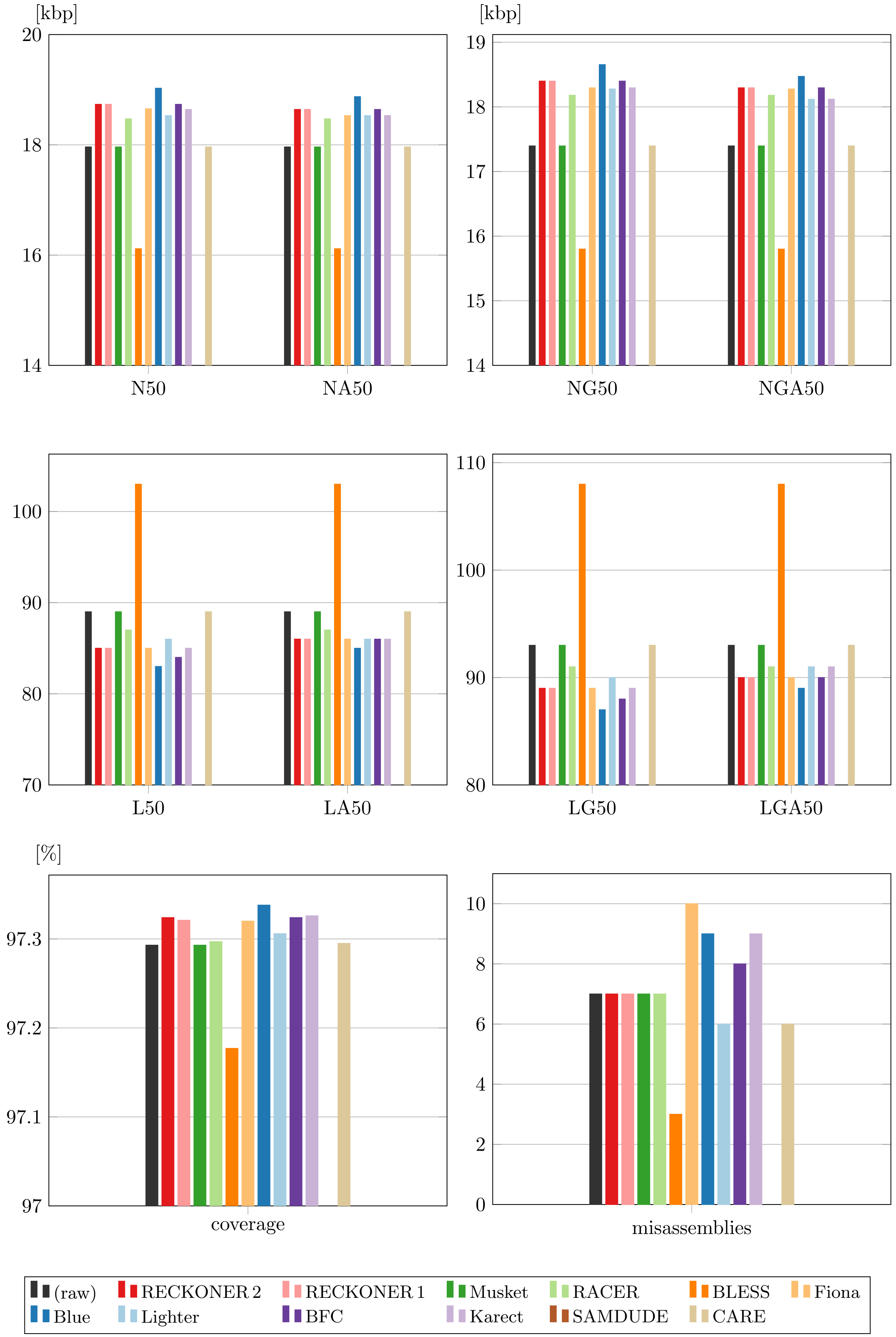

Figure 11: Assembly quality of MiSeq *P. syringae* reads — Minia

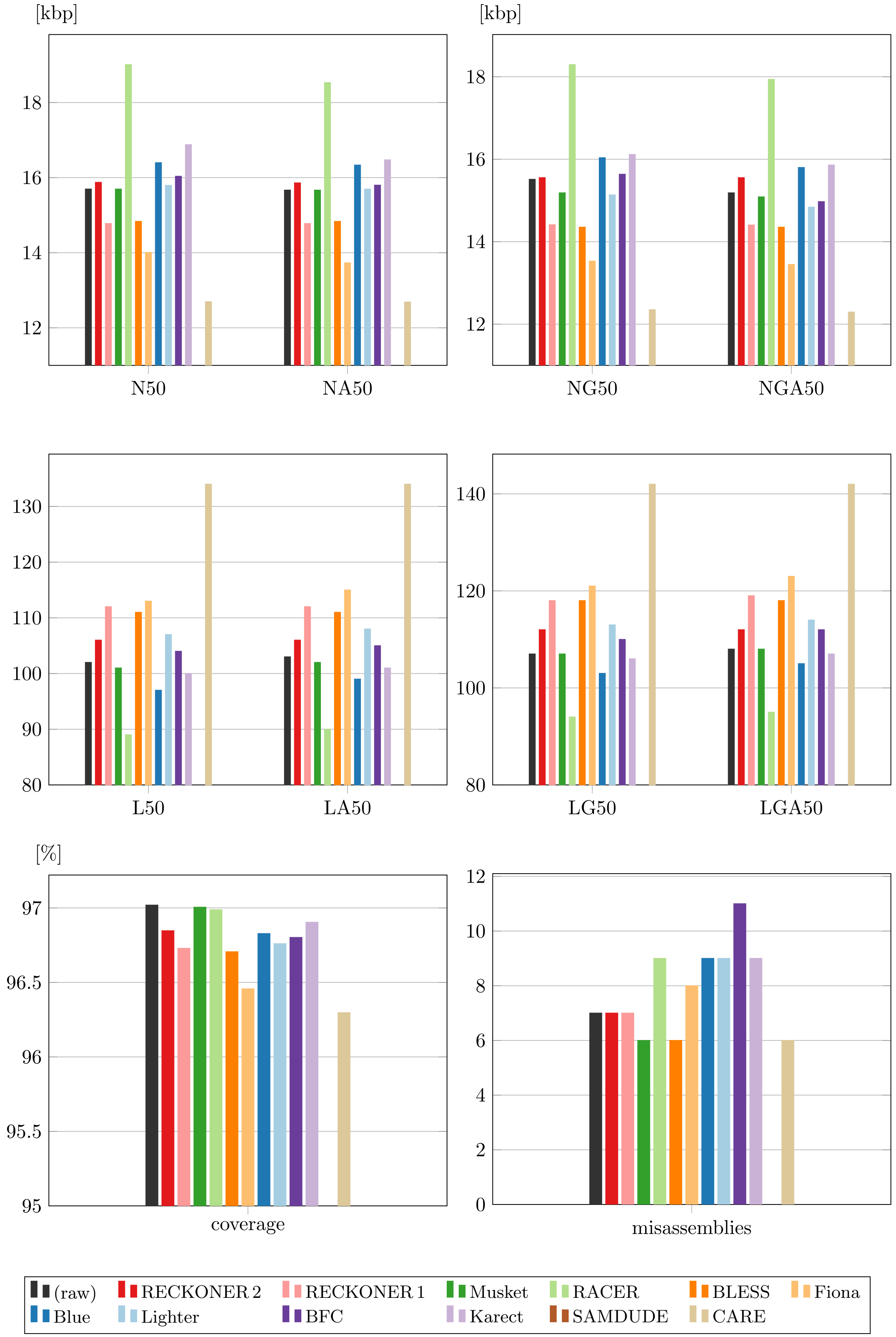

Figure 12: Assembly quality of MiSeq *P. syringae* reads — Velvet

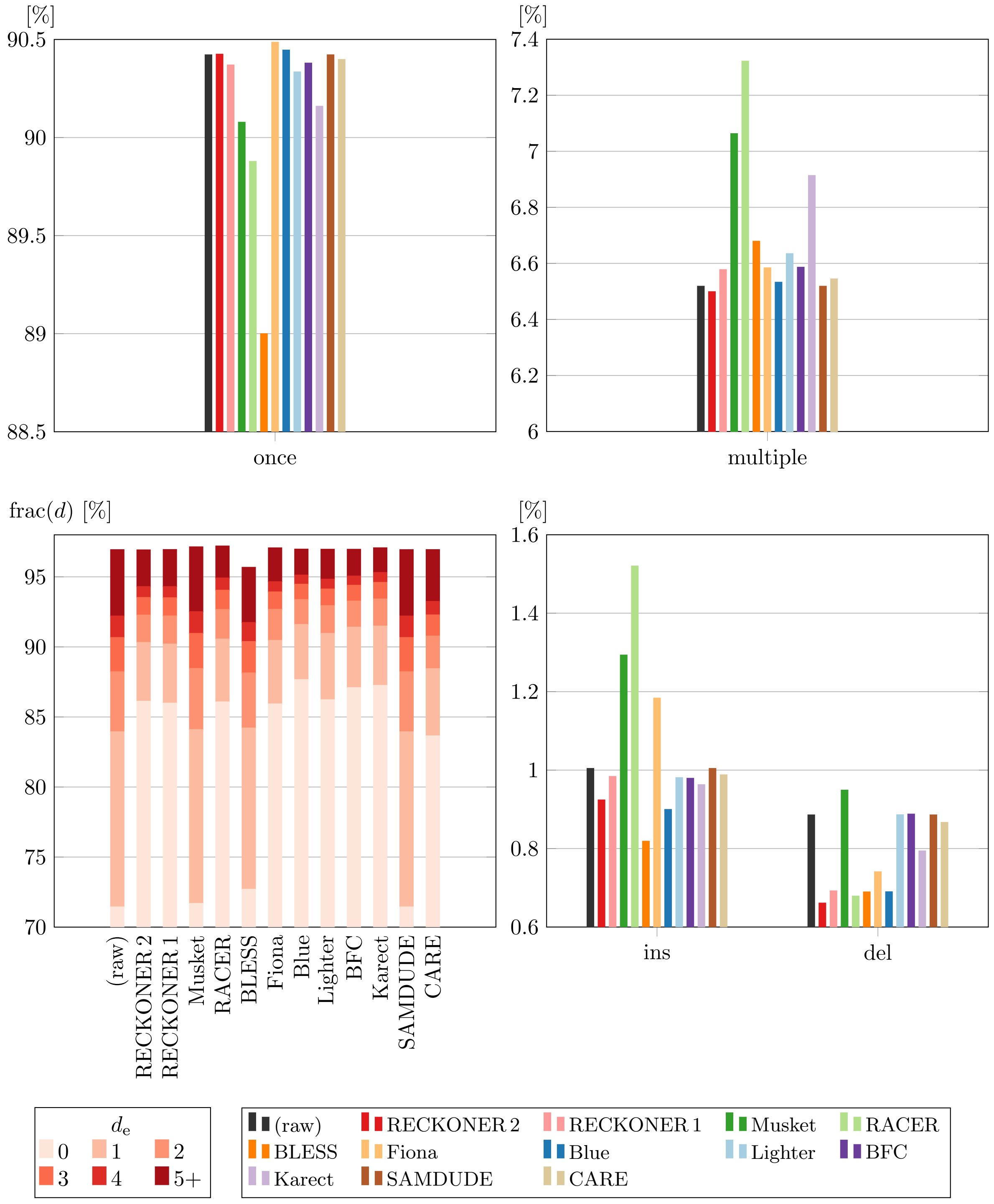

Figure 13: Mapping quality of NovaSeq *C. vulgaris* reads — BWA-MEM

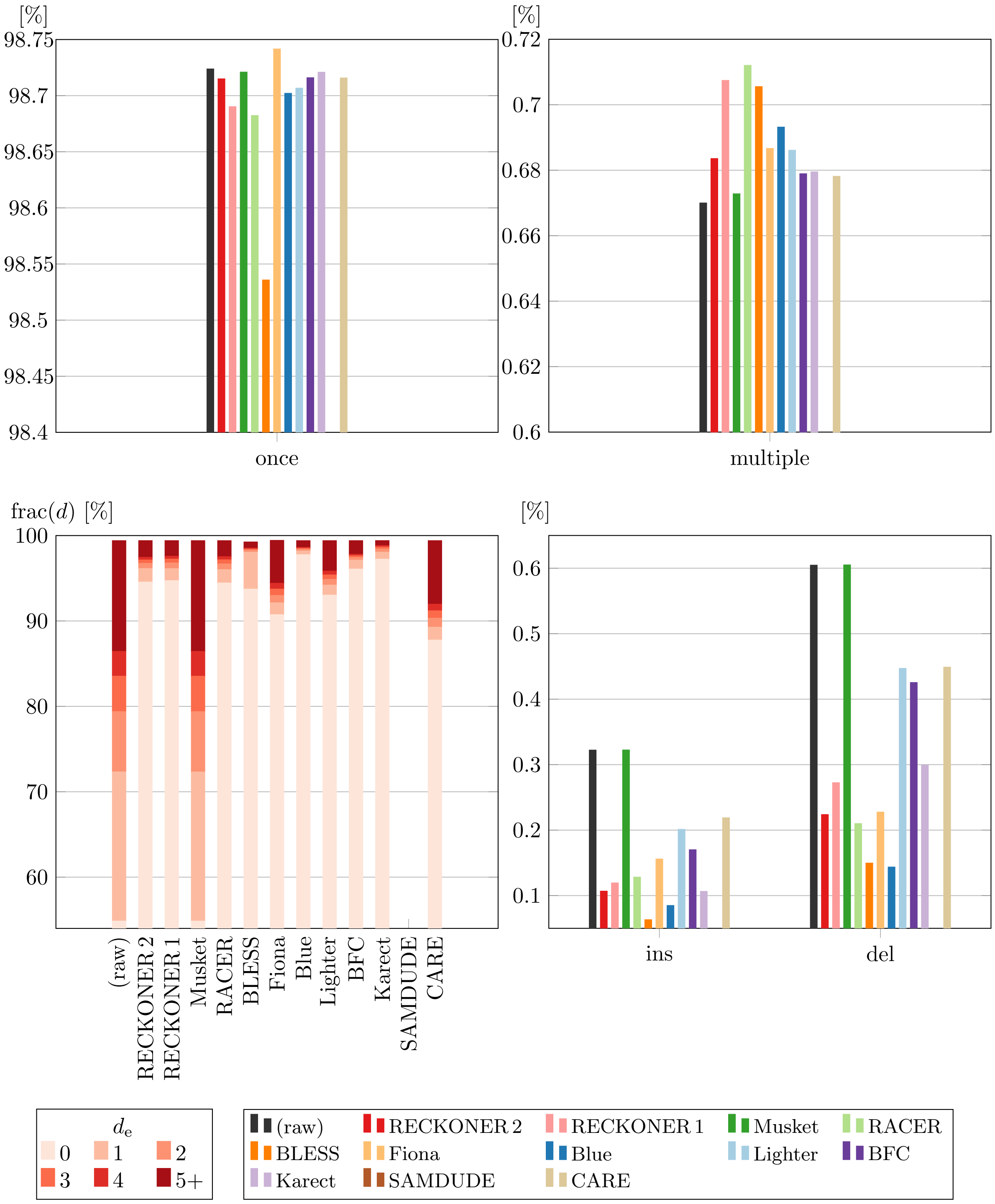

Figure 14: Mapping quality of NovaSeq *P. syringae* reads — BWA-MEM

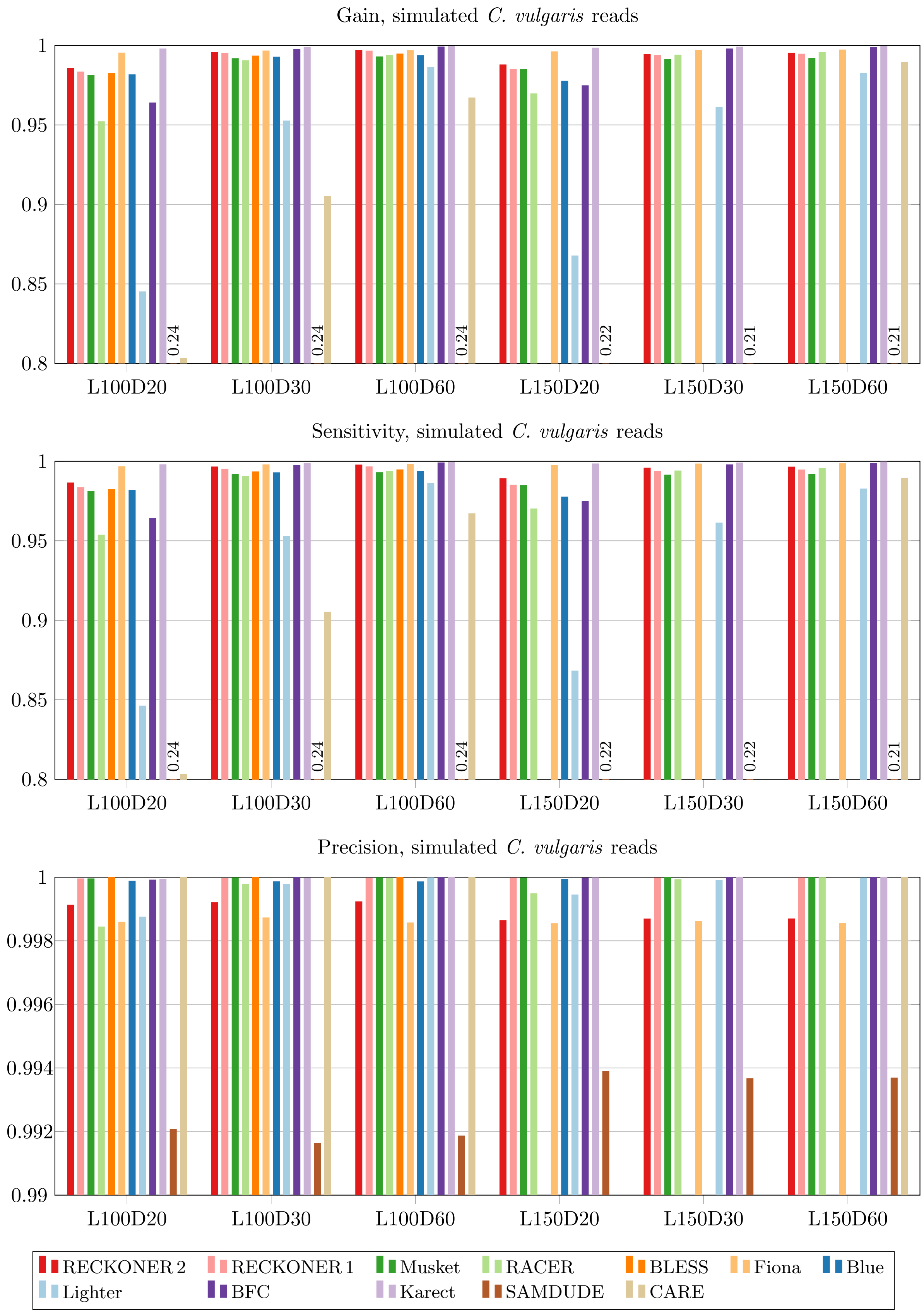

Figure 15: Results for *C. vulgaris* simulated reads — ART

2 Cases studies

2.1 NovaSeq reads characteristics impact

We examined a correction efficacy on NovaSeq reads. The reads may have the following different characteristics in contrast to a popular HiSeq instruments [1]:

- quality binning — number of possible values of symbols a quality scores is reduced to 4 (in contrast to 8 or about 40 in older machines). Correctors may be affected when they rely detection or correction strategies on scores values, what is a case in some algorithms; in the other algorithms we have lack of information about quality scores utilization. Moreover, the binning may be especially important, when the algorithm rely just on quality scores to detect erroneous regions in the read, as it was done in Trowel [4] (discussed in one of our previous work [2]);
- overestimated quality scores — probabilities of substitution errors coded in quality scores are lower than the real error rates. Correctors may be affected under circumstances as in the above point;
- different, depending on position substitution rates of different errors — probability of substitution between a specified symbol and another one is not constant, but depends on values symbols and position in the read. Correctors may be affected, when an assumption about unique characteristics of different read regions is made. Generally, it is a case in all of the correctors.

Table 1 shows a potential vulnerability of different correctors on those traits.

Table 1: Correctors vulnerability on NovaSeq characteristics

Trait	RECKONER 2	RECKONER 1	Musket	RACER
Binning	yes	yes	lack of information	lack of information
Overestimating	yes	yes	lack of information	lack of information
Rates variability	yes	yes	yes	yes

Trait	BLESS	Fiona	Blue	Lighter
Binning	yes	lack of information	lack of information	yes
Overestimating	yes	lack of information	lack of information	yes
Rates variability	yes	yes	yes	yes

Trait	BFC	Karect	SAMDUDE	CARE
Binning	lack of information	lack of information	yes	yes
Overestimating	lack of information	lack of information	yes	yes
Rates variability	yes	yes	yes	yes

2.2 Correction impact on variant calling

On figs. 16 to 21 we posted IGV [5] visualizations of selected loci in human chromosome 1, comparing reads mapped there. Diagrams show that it is not obvious, how correction may impact eliminating of a wrong variant or introducing one (upper diagrams correspond to raw, lower correspond to corrected with RECKONER 2 reads).

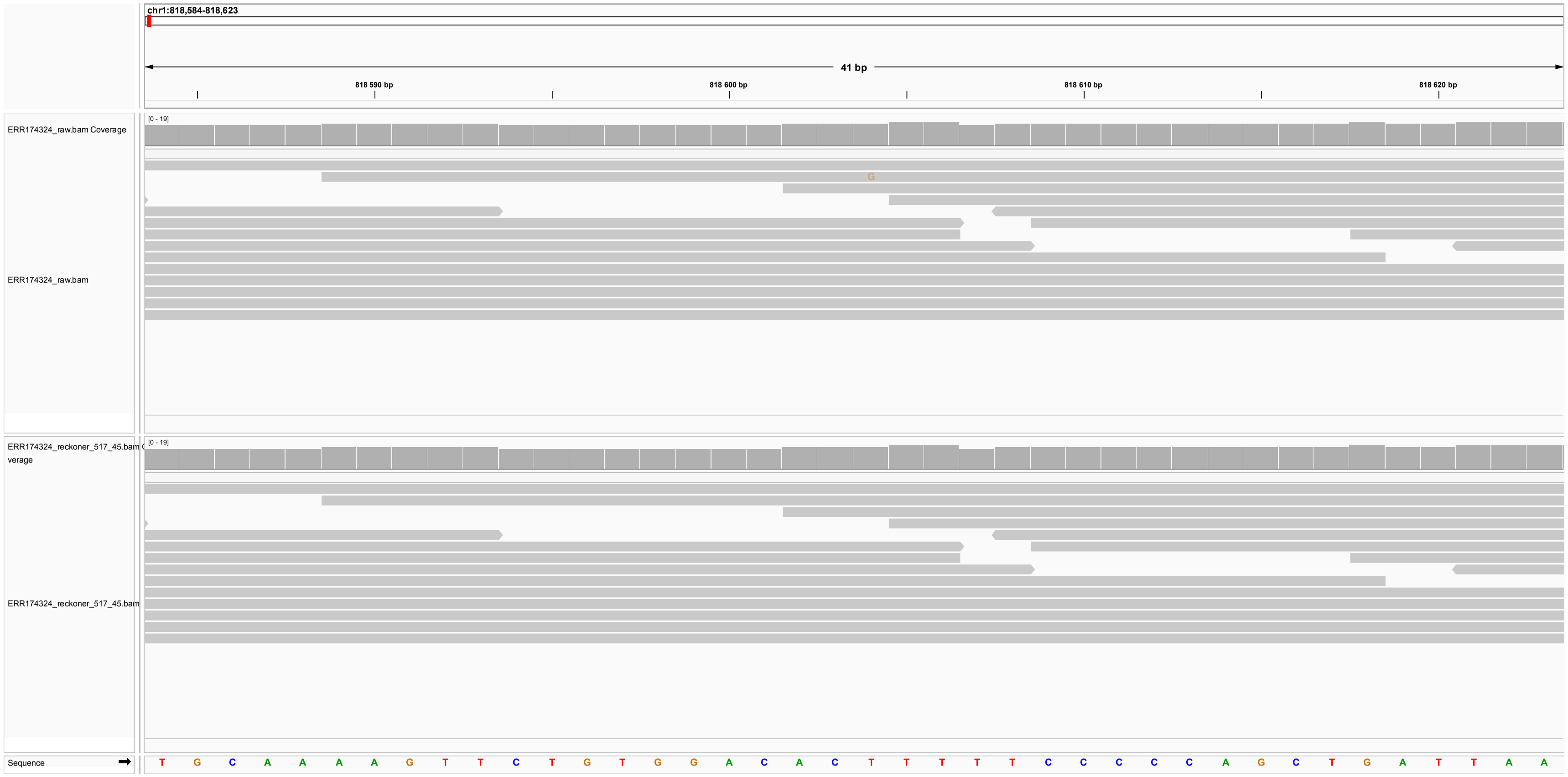

Figure 16: ERR174324, chromosome 1, locus 818,604, site of FP SNP corrected by RECKONER 2. A single G symbol in the uncorrected reads caused a false variant detection, its successfull correction caused the variant to disappear.

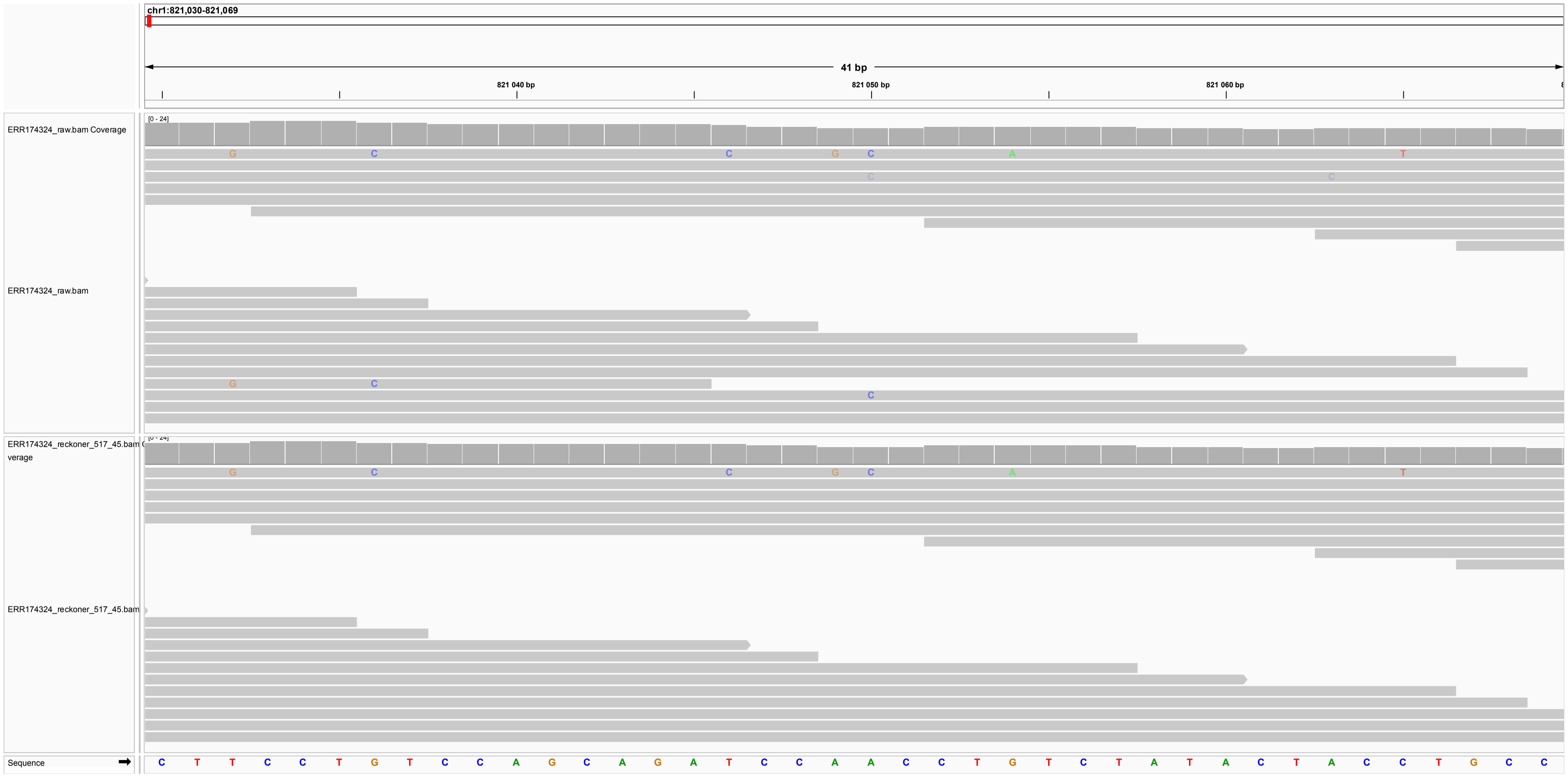

Figure 17: ERR174324, chromosome 1, locus 821,050, site of FP SNP corrected by RECKONER 2. Three C symbols in the uncorrected reads caused a false variant detection, successfull correction of two of them caused the variant to disappear.

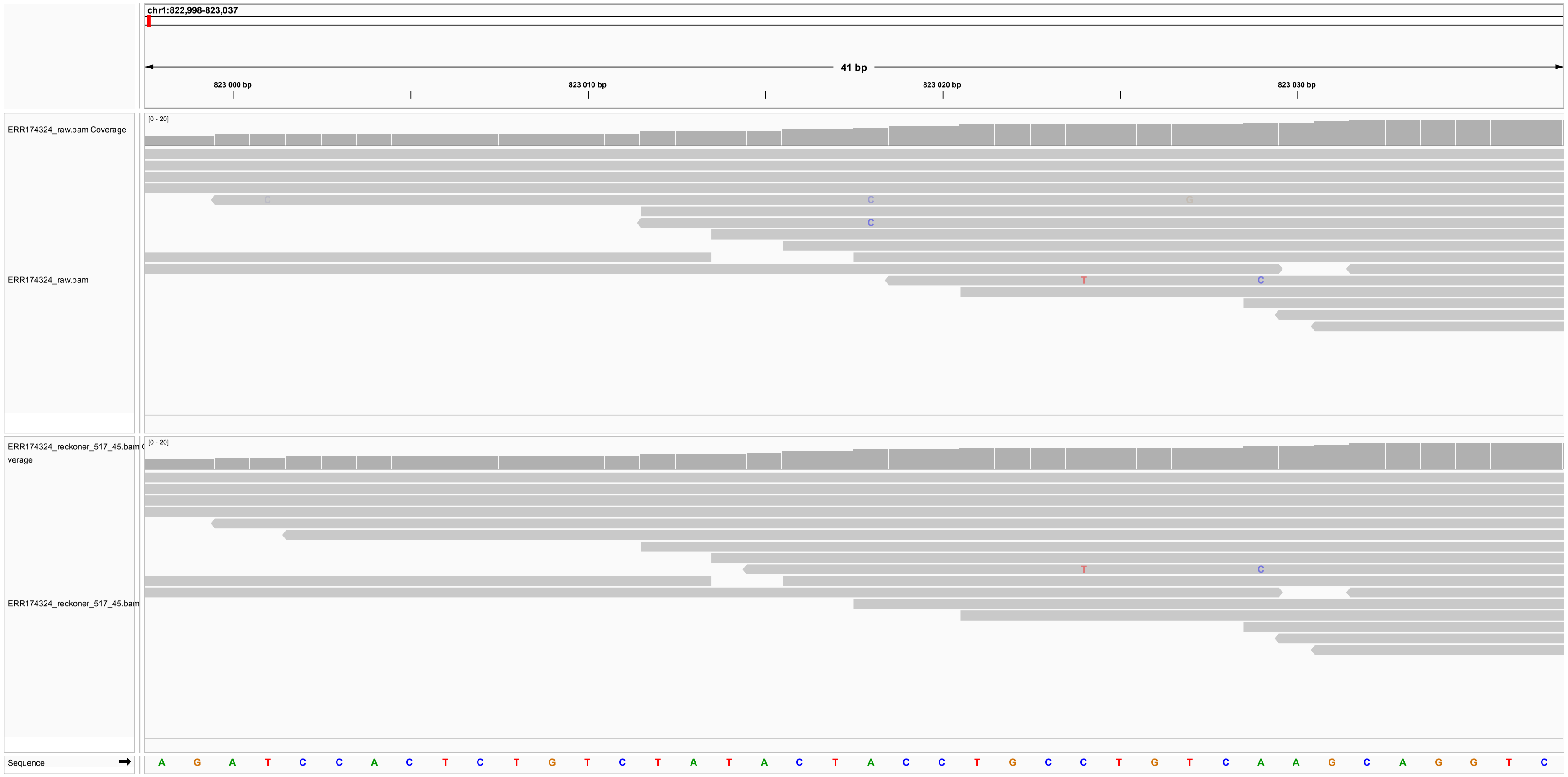

Figure 18: ERR174324, chromosome 1, locus 823,018, site of FP SNP corrected by RECKONER 2. Two C symbols in the uncorrected reads caused a false variant detection, successfull correction of them caused slightly another set of the reads to map in the locus, and caused the variant to disappear.

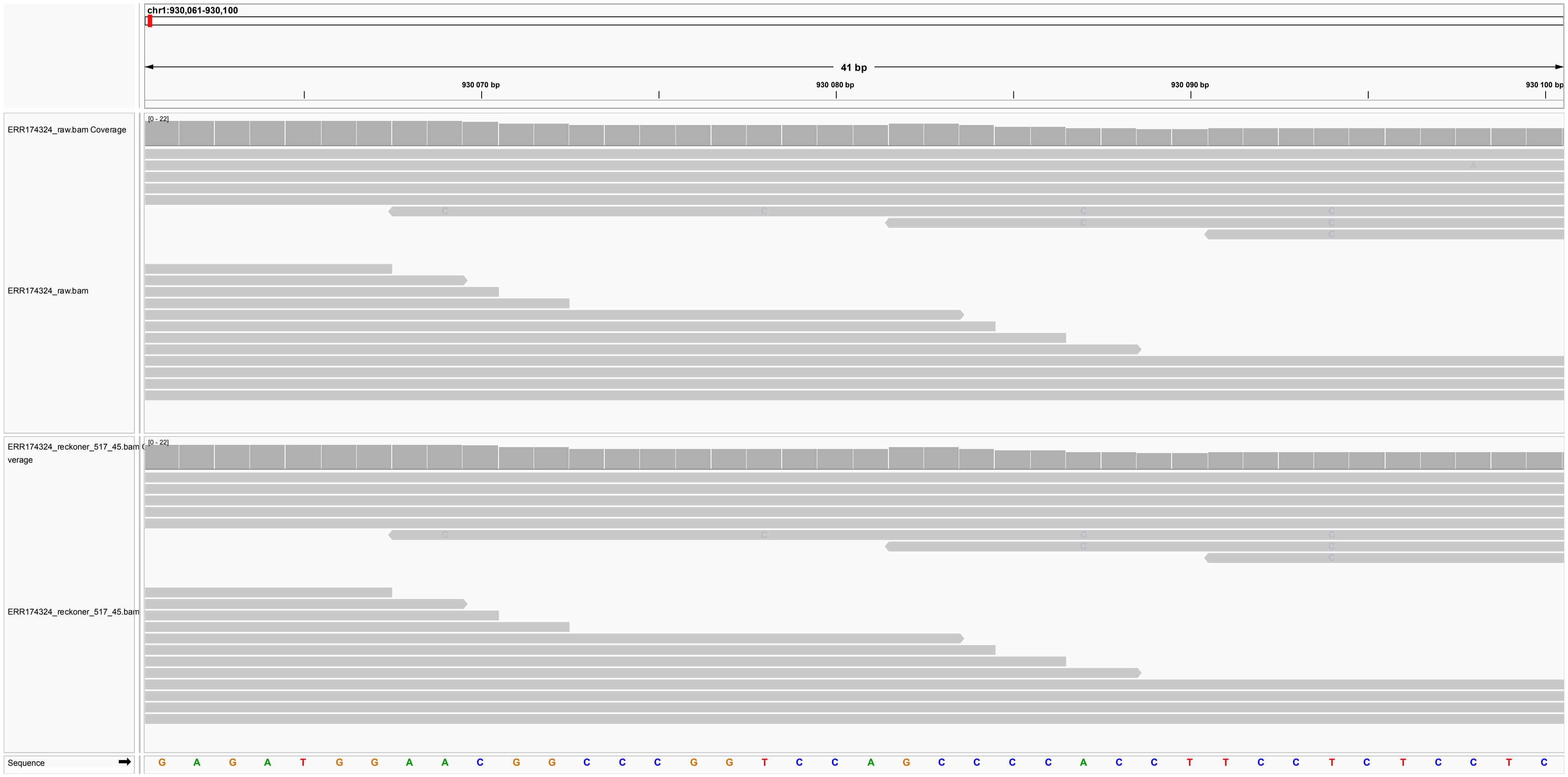

Figure 19: ERR174324, chromosome 1, locus 930,081, site of FP indel introduced by RECKONER 2. After correction nothing visible changed in a group of reads mapped to the locus, but a new false variant was called.

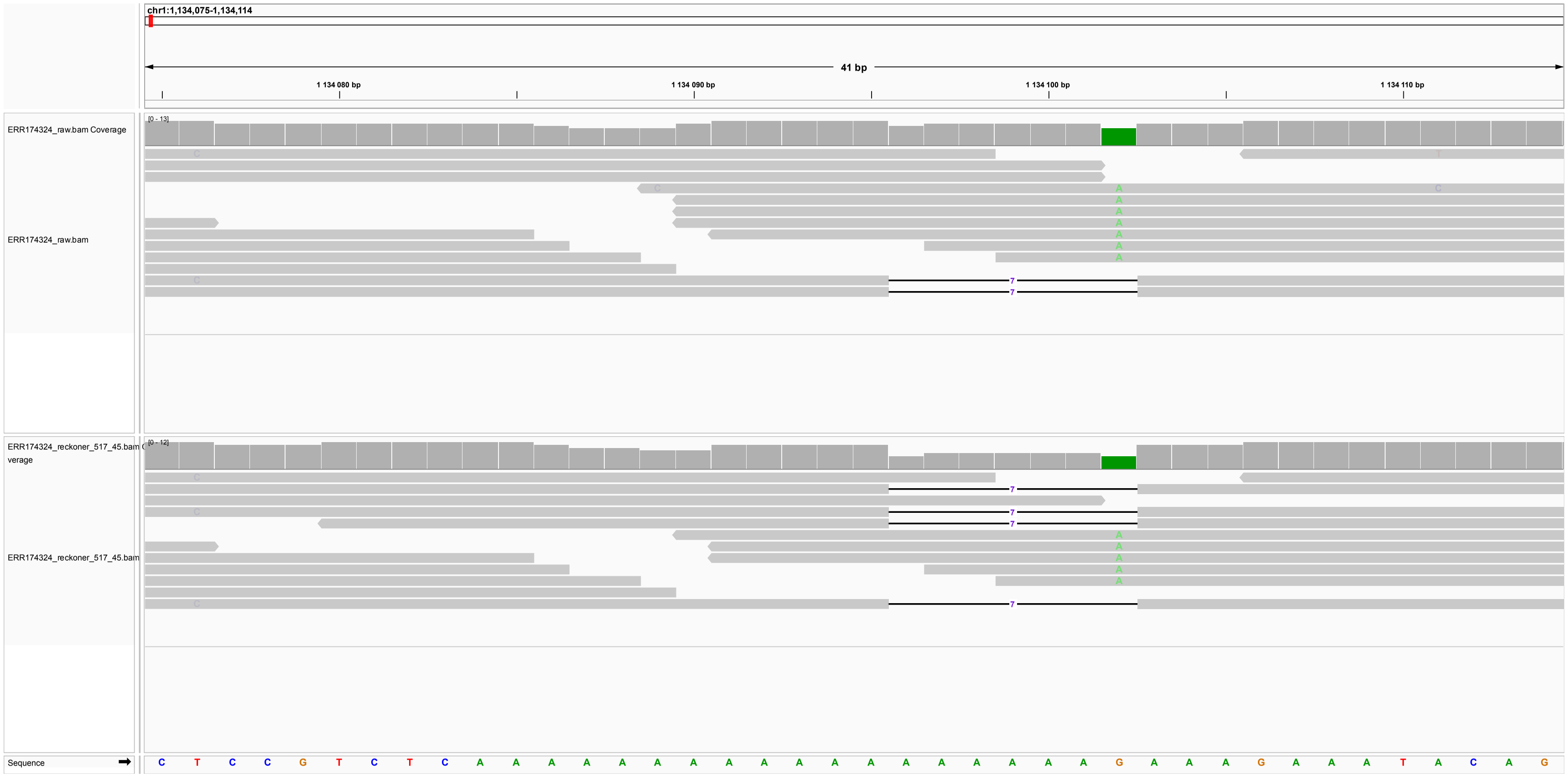

Figure 20: ERR174324, chromosome 1, locus 1,134,095, site of FP indel introduced by RECKONER 2. After correction another set of reads mapped to the locus, causing a new false variant to be called. However, that variant is also supported by another two reads, which earlier were mapped to the locus.

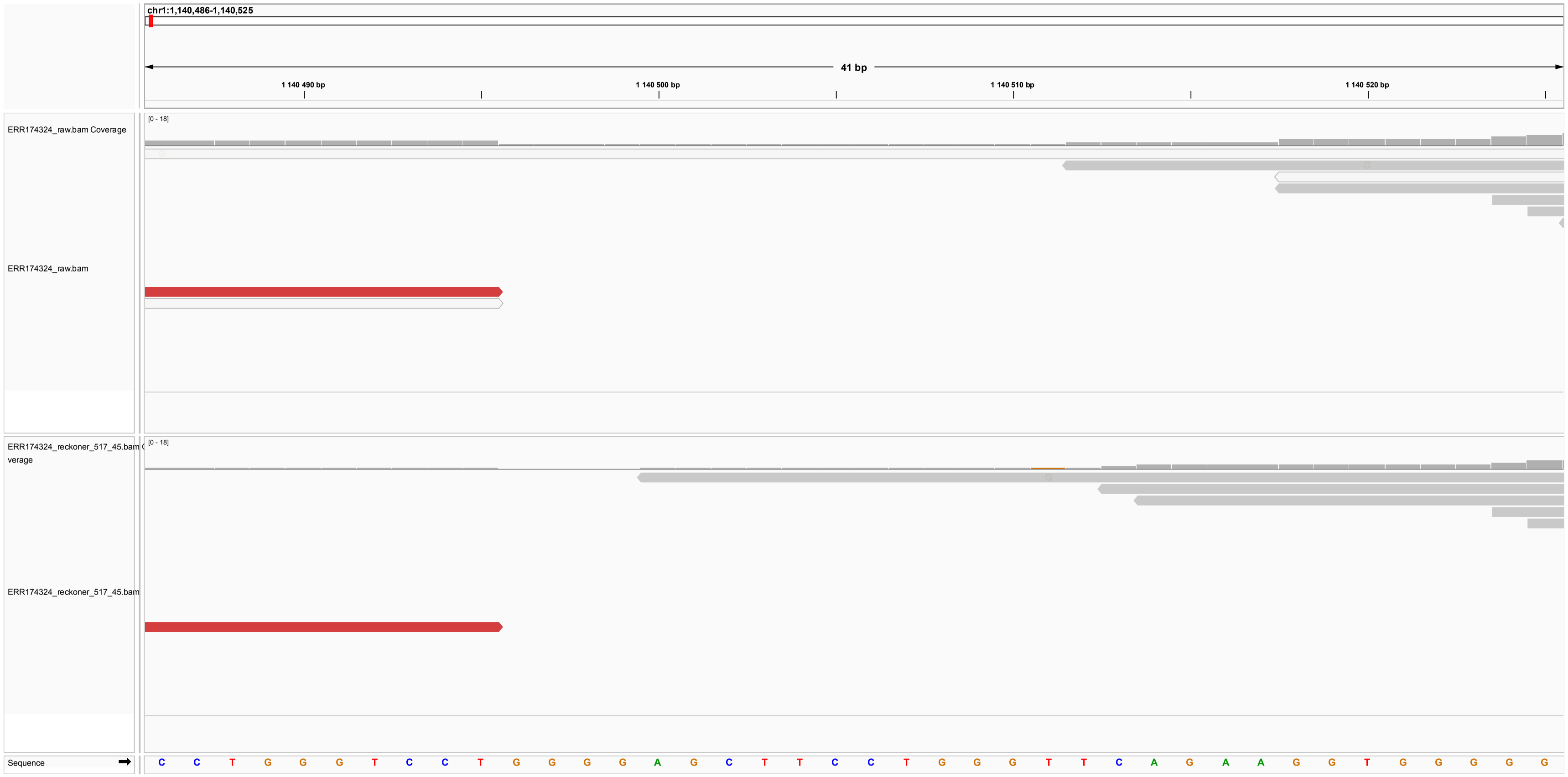

Figure 21: ERR174324, chromosome 1, locus 1,140,506, site of FP indel introduced by RECKONER 2. After correction a single read mapped to the locus, causing a new false variant to be called. However, the read seems to have a sequence identical with the genome.

3 Data sources

The reads were downloaded from:
ftp://ftp.sra.ebi.ac.uk/vol1/fastq/ERR174/ERR174324/ERR174324_1.fastq.gz
ftp://ftp.sra.ebi.ac.uk/vol1/fastq/ERR174/ERR174324/ERR174324_2.fastq.gz
ftp://ftp.sra.ebi.ac.uk/vol1/fastq/ERR174/ERR174325/ERR174325_1.fastq.gz
ftp://ftp.sra.ebi.ac.uk/vol1/fastq/ERR174/ERR174325/ERR174325_2.fastq.gz
ftp://ftp.sra.ebi.ac.uk/vol1/fastq/ERR174/ERR174326/ERR174326_1.fastq.gz
ftp://ftp.sra.ebi.ac.uk/vol1/fastq/ERR174/ERR174326/ERR174326_2.fastq.gz
ftp://ftp.sra.ebi.ac.uk/vol1/fastq/ERR174/ERR174327/ERR174327_1.fastq.gz
ftp://ftp.sra.ebi.ac.uk/vol1/fastq/ERR174/ERR174327/ERR174327_2.fastq.gz
ftp://ftp.sra.ebi.ac.uk/vol1/fastq/ERR134/006/ERR1341796/ERR1341796_1.fastq.gz
ftp://ftp.sra.ebi.ac.uk/vol1/fastq/ERR134/006/ERR1341796/ERR1341796_2.fastq.gz
ftp://ftp.sra.ebi.ac.uk/vol1/fastq/SRR194/004/SRR1945754/SRR1945754_1.fastq.gz
ftp://ftp.sra.ebi.ac.uk/vol1/fastq/SRR194/004/SRR1945754/SRR1945754_2.fastq.gz
ftp://ftp.sra.ebi.ac.uk/vol1/fastq/SRR947/007/SRR9478717/SRR9478717_1.fastq.gz
ftp://ftp.sra.ebi.ac.uk/vol1/fastq/SRR947/007/SRR9478717/SRR9478717_2.fastq.gz
ftp://ftp.sra.ebi.ac.uk/vol1/fastq/SRR111/002/SRR1119292/SRR1119292_1.fastq.gz
ftp://ftp.sra.ebi.ac.uk/vol1/fastq/SRR111/002/SRR1119292/SRR1119292_2.fastq.gz
ftp://ftp.sra.ebi.ac.uk/vol1/fastq/DRR031/DRR031158/DRR031158_1.fastq.gz
ftp://ftp.sra.ebi.ac.uk/vol1/fastq/SRR180/008/SRR1802178/SRR1802178_1.fastq.gz
ftp://ftp.sra.ebi.ac.uk/vol1/fastq/SRR065/SRR065390/SRR065390_1.fastq.gz
ftp://ftp.sra.ebi.ac.uk/vol1/fastq/SRR650/SRR650760/SRR650760_1.fastq.gz
ftp://ftp.sra.ebi.ac.uk/vol1/fastq/DRR031/DRR031158/DRR031158_1.fastq.gz
ftp://ftp.sra.ebi.ac.uk/vol1/fastq/SRR180/008/SRR1802178/SRR1802178_1.fastq.gz

Reads characteristics are shown in Table 2.

Table 2: Read datasets

Accession number	Instrument	Sequencing depth
ERR174324	HiSeq 2000	16
ERR174324, ERR174325	HiSeq 2000	31
ERR174324, ERR174325, ERR174326	HiSeq 2000	47
ERR174324, ERR174325, ERR174326, ERR174327	HiSeq 2000	62
ERR1341796	HiSeq X Ten	55, reduced to $15, 30, 45, 55$
SRR1945754	HiSeq 2000	181, reduced to $10, 20, \ldots, 100$
SRR9478717	NovaSeq 6000	294, reduced to 60
SRR1119292	MiSeq	106, reduced to 60
DRR031158	HiSeq 2500	not relevant
SRR1802178	HiSeq 2000	not relevant

The reference genomes were downloaded from National Library of Medicine (https://www.ncbi.nlm.nih.gov/). FASTA file headers of the *A. thaliana* genome were modified by replacing them with a consecutive natural numbers. It allowed to keep a technical consistency with a ground truth file.

The ground truth sets for human variants were downloaded from:
ftp://ftp-trace.ncbi.nlm.nih.gov/giab/ftp/release/NA12878_HG001/NISTv3.3.2/GRCh38/HG001_GRCh38_GIAB_highconf_CG-IllFB-IllGATKHC-Ion-10X-SOLID_CHROM1-X_v.3.3.2_highconf_PGandRTGphasetransfer.vcf.gz
ftp://ftp-trace.ncbi.nlm.nih.gov/giab/ftp/release/NA12878_HG001/NISTv3.3.2/GRCh38/HG001_GRCh38_GIAB_highconf_CG-IllFB-IllGATKHC-Ion-10X-SOLID_CHROM1-X_v.3.3.2_highconf_PGandRTGphasetransfer.vcf.gz.tbi
ftp://ftp-trace.ncbi.nlm.nih.gov/giab/ftp/release/NA12878_HG001/NISTv3.3.2/GRCh38/HG001_GRCh38_GIAB_highconf_CG-IllFB-IllGATKHC-Ion-10X-SOLID_CHROM1-X_v.3.3.2_highconf_nosomaticdel_noCENorHET7.bed

The ground truth sets for *A. thaliana* variants were downloaded from:
https://1001genomes.org/data/GMI-MPI/releases/v3.1/intersection_snp_short_indel_vcf/intersection_6904.vcf.gz

As the ground truth sets for human variants evaluated with Syndip we used the set available with Syndip itself.

Table 3 shows accession numbers of read datasets, reference genomes identifiers and versions of ground truth VC sets used in the experiments . The genome lengths were `expected_ungapped_length` values from the NCBI database: https://api.ncbi.nlm.nih.gov/genome/v0/expected_genome_size?species_taxid=, where the `taxid` values are shown in Table 4.

Table 3: Read datasets and genomes used in the experiments not shown in the main article

Organism	Reads accession number	Reference genome	VC ground truth
Homo sapiens (for Syndip)	ERR1341796	GRCh38	Syndip
Pseudomonas syringae	SRR1119292	GCF_000585725.1	—

Table 4: Reference genomes

Organism	Genome length	NCBI taxid
H. sapiens	2.823 Gbp	9606
A. thaliana	119.2 Mbp	3702
C. vulgaris	37.73 Mbp	3077
P. syringae	6.034 Mbp	317

4 Experiments details

If possible, all the algorithms were parametrized with gzipped paired reads. When the algorithm was not supporting paired reads, we concatenated the input files, passed them to the algorithm and the splitted the output file. If the algorithm was not supporting gzipped input, we passed decompressed ones.

4.1 Algorithms versions

Table 5 presents versions of correctors used in the experiments. The other used algorithms were shown in Table 6.

4.2 Running the correction algorithms

The algorithms parameters were:

- <alpha> — probability α of selecting a k-mer (Lighter),
- <depth> — sequencing depth,
- <cutoff> — cutoff threshold,
- <fastq_in>, <fastq_in1>, <fastq_in2> — input FASTQ files (respectively: non-paired reads file, paired reads files),
- <fastq_out>, <fastq_out1>, <fastq_out2> — output FASTQ files (respectively: non-paired reads file, paired reads files),
- <fastq_out_pref> — output file prefix (BLESS),
- <genome> — reference genome FASTA file,
- <k> — k-mer length,
- <kmers> — number of k-mers in the input reads,
- <memory> — RAM usage limit (250 for Karect, 250G for CARE),
- <name> — file name core,
- <ploidy> — ploidy (haploid for *C. vulgaris*; diploid for *H. sapiens* and *A. thaliana* — Karect),
- <sam_in> — input SAM file (SAMDUDE),
- <sam_out> — output SAM file (SAMDUDE),
- <threads> — number of threads (48).

The measurable values were chosen as follows:

- <alpha> — as the authors specified, the value should be inversely proportional to sequencing depth and for depth 70 should be 0.1, so the value equals $\frac{7}{\texttt{<depth>}}$,
- <cutoff> — the value of cutoff threshold generated by RECKONER,
- <kmers> — the value „No. of unique k-mers" returned by KMC [3].

RECKONER

RECKONER 1 (all executions) and RECKONER 2 (for variant calling):

```
reckoner -kmerlength <k> -threads <threads> <fastq_in1> <fastq_in2>
```

RECKONER 2 for *de novo* assembly:

```
reckoner -longkmer -kmerlength <k> -threads <threads> <fastq_in1> <fastq_in2>
```

Musket

```
musket -k <k> <kmers> -p <threads> -inorder -omulti <name> <fastq_in1> <fastq_in2>
```

RACER

```
RACER_Linux_parallel <fastq_in> <fastq_out> <genome>
```

BLESS

```
bless -kmerlength <k> -read1 <fastq_in1> -read2 <fastq_in2> -smpthread <threads> \
-prefix <fastq_in_pref> -gzip -max_mem 8
```

Fiona

```
fiona -nt <threads> -g <genome> <fastq_in> <fastq_out>
```

Blue

```
mono Tessel.exe -k <k> -g <genome> -t <threads> -f fastq -tmp .  <name> <fastq_in1> <fastq_in2>
mono GenerateMerPairs.exe -t <threads> <name>.cbt <fastq_in1> <fastq_in2>
mono Blue.exe -m <cutoff> -t <threads> -r o -o .  -f fastq <name>.cbt <fastq_in1> <fastq_in2>
```

Lighter

```
lighter -r <fastq_in1> -r <fastq_in2> -k <k> <genome> <alpha> -t <threads>
```

BFC

```
bfc -s <genome> -t <threads> <fastq_in> > <fastq_out>
```

Karect

```
karect -correct -threads=<threads>-memory=<memory> -celltype=<ploidy> \
-matchtype=hamming -inputfile=<fastq_in1> -inputfile=<fastq_in2>
```

SAMDUDE

```
python3 run_denoiser.py <sam_in> <sam_out>
```

CARE

```
care-cpu -d .  -c 7/<coverage> -i <fastq_in1> -i <fastq_in2> -o <fastq_out1> \
-o <fastq_out2> --pairmode PE -t <threads> -m <memory> --candidateCorrection -q \
--excludeAmbiguous
```

4.3 Running the other algorithms and pipelines

The algorithms parameters were:

- `<bam>` — sorted BAM file,
- `<bam_dir>` — working directory (Strelka),
- `<bam_raw>` — non-sorted BAM file,
- `<bed>` — confident call regions file,
- `<fasta>` — assembly output contig file,
- `<fastq_in>`, `<fastq_in1>`, `<fastq_in2>` — input FASTQ files,

- <fastq_out> — output FASTQ file (ART),
- <gt_vcf> — ground truth file,
- <in_dir> — Docker input directory (Deepvariant),
- <length> — reads length,
- <memory> — RAM usage limit (250 for Minia),
- <name> — file name core,
- <num_reads> — number of reads (ART),
- <out_dir> — Docker output directory (Deepvariant),
- <ploidy> — organism ploidy (Karect),
- <quast_out> — assembly evaluation output directory (Quast),
- <ref> — reference genome file,
- <sam> — mapping output file,
- <threads> — number of threads (48).

Genome indexing — BWA

All the reference genomes were initially indexed with BWA:

```
bwa index <ref>
```

Variant calling — Strelka

```
bwa mem -t <threads> -M <ref> <fastq_in1> <fastq_in2> > <sam>
samtools view -@ <threads> -b <sam> > <bam_raw>
samtools sort -@ <threads> <bam_raw> > <bam>
samtools index <bam>
python configureStrelkaGermlineWorkflow.py --bam <bam> --ref <ref> --runDir <bam_dir>
python <bam_dir>/runWorkflow.py -m local -j <threads>
```

Variant calling — Deepvariant

```
(reads mapping as for Strelka)
docker run -v <in_dir>:/input -v <out_dir>/<name>:/output \
google/deepvariant:1.5.0 /opt/deepvariant/bin/run_deepvariant \
--model_type=WGS --ref=/input/<ref> --reads=/input/<bam> \
--output_vcf=/output/<name>.vcf --output_gvcf=/output/<name>.gvcf\
--num_shards=<threads> --logging_dir=/output/<name
```

Variants evaluation — hap.py — *H. sapiens*

```
python hap.py <gt_vcf> <name>.vcf.gz -f <bed> -o <name>.happy.nist -r <ref>
```

Variants evaluation — hap.py — *A. thaliana*

```
python hap.py <gt_vcf> <name>.vcf.gz -o <name>.happy.nist -r <ref>
```

Variants evaluation — Syndip

```
run-flt -o <name> <name>.vcf
run-eval -g 38 <name>.flt.vcf.gz | sh
```

***De novo* assembly — Minia**

```
minia -in <fastq_in1> -in <fastq_in2> -max-memory <memory> -out <fasta>
```

***De novo* assembly — Velvet**

```
velveth .  31 <fastq_in1> -in <fastq_in2>
velvetg .  -cov_cutoff auto
```

Reads mapping — BWA

```
bwa mem -t <threads> -M <ref> <fastq_in1> <fastq_in2> > <sam>
```

Assembly evaluation — Quast

```
quast.py <fasta> -o <quast_out> -R <ref> --threads <threads>
```

Reads mapping (for SAMDUDE) — BWA

```
bwa mem -t <threads> -M <ref> <fastq_in1> <fastq_in2> > <sam>
```

Generating reads profiles for simulation — ART

```
art_profiler_illumina <fastq_in> <name> fastq
```

Reads simulation — ART

```
art_illumina -sam -1 <name> -l <length> -i <ref> -c <num_reads> -o <fastq_out> -rs 0 -na
```

Genotype concordance — GATK

```
gatk Concordance -R <ref> -eval <name>.vcf.gz --truth <gt_vcf> --summary <name>.tsv
```

4.4 Experiments environment

The experiments were performed on a computer equipped with 256 GB of RAM, two Intel(R) Xeon(R) CPU E5-2670 v3 @ 2.30GHz processors, running under Debian 9.3 x86-64 OS. All the algorithms were run with all the resources available in the system (in some cases after specifying `<memory>` or `<threads>` parameters).

4.5 Experiments failures

Human variant calling — Strelka

For human reads utilized for VC evaluated with hap.py (called with Strelka and DeepVariant: combinations of ERR174324, ERR174325, ERR174326, ERR174327) correction failures were as follows. For all the sets Fiona was interrupted with SIGKILL after allocating 250 GiB of memory; Karect timeouted after 72 h; SAMDUDE threw an exception *index is out of bounds*.

For the 15× set Blue was interrupted with Mono message *Got a SIGSEGV while executing native code.* or with a message *Error: Garbage collector could not allocate 16384 bytes of memory for major heap section.*, depending on k. For the 30× set RECKONER 1 for $k = 17$ was interrupted with SIGKILL after allocating 250 GiB; BLESS for tiny oligomer lengths $k = 11$ and $k = 13$ returned a message *ERROR: KMC is abnormally terminated*; Blue finished as for 15×. For the 45× set BLESS for tiny oligomer lengths $k = 13$ and $k = 15$ returned a message as for 30×; Blue finished as for 15×, except for $k = 31$, where the correction succeeded; CARE was interrupted with SIGKILL after allocating 250 GiB of memory. For the 60× set BLESS for tiny oligomer lengths $k = 13$ and $k = 15$ returned a message as for 30×; Blue finished as for 15× or was interrupted after allocating 240 GiB of memory; CARE returned a message *Cannot construct a single cpu hashtable. Abort!*.

Human variant calling — DeepVariant

In these experiment we utilized reads corrected as in the previous subsection, however DeepVariant returned an error *ValueError: DATA_LOSS: Failed to parse SAM record* while processing reads of 45× set corrected with Blue.

Human variant calling — Syndip

For human reads utilized for VC evaluated with Syndip (called with Strelka: subsets of ERR1341796) correction failures were as follows. For the 15× set Fiona was interrupted with SIGABRT after allocating 240 GiB of memory; Karect and SAMDUDE timeouted after 72 h. For the 30× set RECKONER 1 and RACER were interrupted with SIGKILL after allocating 250 GiB of memory; Fiona was interrupted with SIGABRT after allocating 250 GiB of memory; Karect and SAMDUDE timeouted after 72 h; CARE threw an exception *Not enough memory available for hash tables.* For the 45× set the failures reasons were as for 30×, except for CARE, which threw an exception *std::bad_alloc.* For the 60× set the failures reasons were as for 30×, except for BLESS, which returned a message *ERROR: KMC is abnormally terminated*, and CARE, which returned a message *Cannot construct a single cpu hashtable. Abort!*. Blue for 15×, 30×, 55× was not run due to failures of the abovementioned experiments. For 45× it was interrupted with Mono message *Got a SIGSEGV while executing native code.*.

***A. thaliana* VC**

For *A. thaliana* (SRR1945754) reads all the algorithms were able to perform the correction, however, as for human reads in the main article, Fiona changed read quality scores causing callers to generate almost no variants.

***C. vulgaris* NovaSeq reads — assembly and mapping**

For NovaSeq *C. vulgaris* (SRR9478717) reads all the algorithms were able to perform the correction.

***P. syringae* MiSeq reads — assembly and mapping**

For MiSeq *P. syringae* (SRR1119292) SAMDUDE threw an exception *index is out of bounds.*

***C. vulgaris* simulated reads**

For the sets with read lengths 150 bp BLESS reported an error *ERROR: Irregular quality score range 36-75.* For the set L100C20 for $k = 20$ and $k = 22$ and the sets L150C30 L150C60 Blue threw an exception *Index was outside the bounds of the array.*. For the sets L150C20 and L150C30 CARE reported an error *Assertion 'batchReadIds.size() % 2 == 0' failed.*.

Table 5: Correction algorithms versions

Algorithms	Version	Source
RECKONER	2.1	https://github.com/refresh-bio/RECKONER
RECKONER	1.2	https://github.com/refresh-bio/RECKONER
BLESS	1.02	https://sourceforge.net/projects/bless-ec/files/
Musket	1.1	http://musket.sourceforge.net/homepage.htm
RACER	—	https://www.csd.uwo.ca/~ilie/RACER/
Fiona	2.4.0	https://github.com/seqan/seqan
Blue	1.1.3	https://bioinformatics.csiro.au/public/files/
Lighter	1.1.2	https://github.com/mourisl/Lighter/releases
BFC	BFC-ht, version r181	https://github.com/lh3/bfc
Karect	1.0	https://github.com/aminallam/karect
SAMDUDE	version of 2nd May, 2018	https://github.com/irenatfh/SAMDUDE
CARE	2.1	https://github.com/fkallen/CARE

Table 6: Other algorithms versions

Algorithm	Utilization	Version	Source
BWA	Reads mapping	0.7.15-r1140	https://github.com/lh3/bwa
Deepvariant	Variant calling	1.5.0	https://github.com/google/deepvariant
hap.py	Variants evaluation	0.3.9	https://github.com/Illumina/hap.py/releases
Minia	*De novo* assembly	3.2.4	https://github.com/GATB/minia
Quast	Assembly evaluation	5.0.2	https://sourceforge.net/projects/quast/files
SAMtools	SAM files processing	1.9	https://github.com/samtools/samtools
Strelka	Variant calling	2.9.9, centos6_x86_64	https://github.com/Illumina/strelka/releases
Syndip and run-flt	Variants evaluation	0.4	https://github.com/lh3/CHM-eval
Velvet	*De novo* assembly	1.2.10	https://github.com/dzerbino/velvet
ART	Reads simulation	2.5.8	https://www.niehs.nih.gov/research/resources/software/biostatistics/art/index.cfm
GATK	Genotype concordance	4.4.0.0	https://github.com/broadinstitute/gatk/releases

5 Exact algorithms parameters

Tables 7 and 8 shows k-mer lengths chosen for correctors. The values were selected to obtain the best results for the experiment, however, for experiments with Syndip we used the parameters obtained for experiments with hap.py.

Table 7: Correctors oligomer length (k) for VC

Algorithm	*A. thaliana*										*H. sapiens*			
	10×	20×	30×	40×	50×	60×	70×	80×	90×	100×	15×	30×	45×	60×
RECKONER 2	21	21	21	25	25	25	25	23	21	21	59	59	59	59
RECKONER 1	21	21	23	23	17	17	17	17	17	17	41	19	23	23
Musket	27	27	27	27	27	27	27	27	27	27	13	13	13	23
BLESS	39	23	25	35	37	37	41	29	39	29	21	15	17	17
Blue	21	31	31	31	31	31	31	31	31	31	—	—	—	—
Lighter	31	31	31	31	29	31	31	31	31	29	11	13	11	11

Table 8: Correctors oligomer length (k) for *de novo* assembly, reads mapping and simulated reads

Algorithm	*C. vulgaris*	*P. syringae*	*C. vulgaris* (simulated)
RECKONER 2	37	21	22
RECKONER 1	36	22	21
Musket	27	27	23
BLESS	25	18	21
Blue	31	29	23
Lighter	32	31	20

References

[1] On NovaSeq Base Quality. https://lh3.github.io/2017/07/24/on-nonvaseq-base-quality. [accessed: November 19, 2023].

[2] M. Długosz and S. Deorowicz. RECKONER: read error corrector based on KMC. *Bioinformatics*, 33(7):1086–1089, 2017.

[3] M. Kokot, M. Długosz, and S. Deorowicz. KMC 3: counting and manipulating k-mer statistics. *Bioinformatics*, 33(17):2759–2761, 2017.

[4] E.-C. Lim, J. Müller, J. Hagmann, S.R. Henz, S.-T. Kim, and D. Weigel. Trowel: a fast and accurate error correction module for Illumina sequencing reads. *Bioinformatics*, 30(22):3264–3265, 2014.

[5] H. Thorvaldsdóttir, J.T. Robinson, and J.P. Mesirov. Integrative genomics viewer (igv): high-performance genomics data visualization and exploration. *Briefings in bioinformatics*, 14(2):178–192, 2013.
